# Pilot study on the dynamic interactions between cardiac activity and corneal biomechanics during eye movements

**DOI:** 10.3389/fmed.2024.1484449

**Published:** 2024-12-10

**Authors:** Mohammadali Shahiri, Henryk Kasprzak, Magdalena Asejczyk

**Affiliations:** Department of Optics and Photonics, Wrocław University of Science and Technology, Wrocław, Poland

**Keywords:** corneal deformations, corneal dynamics, fixational eye movements, blood pulsation, eye biomechanics

## Abstract

**Background and objective:**

The study examines the relationship between ocular rotations and cardiovascular functions through detailed biomechanical analysis. The study documents specific patterns of ocular movements and their synchronization with cardiovascular activity, highlighting significant correlations. These findings provide a basis for understanding the opto-biomechanical interplay between ocular and cardiovascular dynamics.

**Methods:**

Authors employed a custom-designed prototype, integrating a camera and numerical pulse oximeter, to analyze the right eyeballs of participants. The corneal surface reflections were recorded, along with concurrent blood pulsation (BP) signal acquisition. Numerical analysis helped determine the reflection positions and orientations to study eye movements and corneal deformations. Time and frequency domain analyses, including coherence functions, were utilized.

**Results:**

Significant correlations were found between variations in corneal curvature, selected fixational eye movements (FEM) parameters, and Pulsatile ocular blood flow (POBF), revealing synchronized expansions of the corneal surfaces with cardiovascular activity. Furthermore, FEM displayed spectral correlations with BP, indicating an interrelation between ocular rotations and cardiovascular functions.

**Conclusion:**

These findings reveal the complex interactions between the cornea and Pulsatile Ocular Blood Flow (POBF), as well as between Fixational Eye Movements (FEM) and POBF. While the influence of POBF on both corneal dynamics and FEM is clear, further research is necessary to directly link corneal dynamics and FEM. These insights hold potential for non-invasive diagnostic applications and provide a deeper understanding of ocular biomechanics.

## 1 Introduction

The cornea, a critical component in the ocular system, is known for its significant optical power and accessibility (1, 2). It displays a range of complex biomechanical and optical properties that are essential for vision. Consequently, there has been a substantial amount of research focused on decoding the biomechanical and optical aspects of the cornea. Despite this, most studies have examined its properties in isolation, overlooking the inherent interconnections. It’s important to recognize that the corneal optical parameters are linked to its geometrical and biomechanical characteristics (3). These characteristics are further influenced by the cornea’s heterogeneous and anisotropic nature, shaped by physiological factors (4). This complexity implies that the mechanical properties of the cornea are distributed in a three-dimensional pattern, akin to what is observed in conditions such as keratoconus (5). Adding to this complexity is the observation that the corneal elastic modulus and other mechanical properties vary along different radial meridians (6, 7). When assessing the cornea, it’s crucial to consider the influence of various external structures and media, particularly intraocular pressure (IOP). For example, an elevation in IOP leads to anterior displacement of the cornea, which in turn alters the axial length of the eye (8). Such changes create a combination of compressive and tensile forces within the cornea, complicating the measurement of its elastic properties (3). Traditionally, the cornea has been viewed as a relatively static element in tonometric examinations. However, it is vital to acknowledge that the cornea is subject to continuous and rapid movements (9). These movements are influenced by deformations stemming from pulsatile fluctuations in IOP, driven by variations in blood pressure (BP) (10–12). These BP changes are characterized by both slow diurnal shifts and rapid, heartbeat-induced fluctuations (10). In light of these complexities, recent research has pivoted toward exploring corneal dynamics *in vivo*. This shift addresses the limitations of both *ex vivo* and *in vivo* studies, including challenges in translating observed tissue behavior to living conditions, potential tissue degradation in *ex vivo* studies, and notable variations between individual eyes in both study types (13–20). These individual differences, such as variations in biomechanical properties and intraocular pressure responses, complicate the generalization of findings across studies.

An area of particular interest is the quasi-periodic nature of eye expansion and its relation to BP. Research in corneal dynamics has highlighted how alterations in IOP and ocular pulse blood flow (POBF) can induce changes in eye shape, such as corneal surface expansion and modifications in eye length (21–24). A key study linked longitudinal corneal apex displacement (LCAD) with pulsatile blood flow (21). This study used a non-invasive approach involving ultrasonic distance sensors to track LCAD and head movements, revealing a correlation between these movements and cardiovascular activity. However, the complex relationship between BP and ocular pulse remains partially understood. Further investigations using high-speed videokeratoscopy have examined how longitudinal eye movements influence corneal shape (25). While these studies identified signals in the movement data related to the pulse, respiration, and blinking, a direct link between longitudinal apex movements and corneal curvature changes is yet to be firmly established.

Additionally, the study of oculomotor movements, predominantly considered as rotational movements of the eye, presents another layer of complexity. While these movements are extensively studied due to their direct impact on visual processes and perception, particularly in relation to saccades and movements over 1 degree, their effect on corneal expansion is less understood. Our research, which involved the use of a standard ophthalmic headrest to stabilize the head during measurements, considered these oculomotor movements. Unexpectedly, detailed analysis of the data obtained revealed spectral correlations with heart activity and BP. Prior studies (26, 27) have observed the influence of such movements on anterior eye measurements, but the spectral correlations of these effects remain under-explored. Our approach, which includes spectral coherence analysis between FEM data and BP, provides new insights into these complex interactions (28, 29). Time-domain analysis alone may overlook underlying periodic patterns and frequency-specific influences, whereas spectral analysis provides insights into the frequency components and dynamic relationships between these physiological signals and eye measurements. It’s crucial to note that even minor head movements can significantly affect ophthalmological measurements (30–32). While often regarded as random, research has shown that these movements exhibit clear spectral correlations with the cardiopulmonary system (33).

In conclusion, understanding the multifaceted nature of the cornea and its interaction with various external factors is vital. In-depth research into *in vivo* corneal dynamics can offer invaluable insights into the cornea’s behavior and its relationship with elements like IOP, POBF, and head movements. This knowledge is crucial for the development of non-invasive diagnostic techniques and enhancing our grasp of the complex mechanisms underlying vision. The present work contributes to this area, specifically focusing on the Finite Element (FE) modeling of the optomechanical self-adjustment mechanism (8), which is a crucial step toward integrating this model with finite element analysis.

## 2 Materials and methods

### 2.1 Participants

This study involved eight healthy volunteers, comprising three females and five males. None of the participants were contact lens users and had no history of dry eye symptoms or any known corneal and heart conditions. Participants were fully informed about the study’s rationale and procedures’ risks. The project was affirmed by the Ethics Committee of the Wroclaw University of Science and Technology (O-22-30) and adhered to the tenets of the Declaration of Helsinki. In this study, the right eyes of the subjects were exclusively examined. Participants were instructed to refrain from blinking for approximately 10 s during each testing session to ensure measurement accuracy. Prior to each session, the instrument was meticulously calibrated to maintain consistent measurement standards. All measurements were conducted by the same experienced investigator, ensuring uniformity in the testing procedure. The experiment was repeated four times for each subject to confirm the reliability and reproducibility of the data. The repetition rate is adopted from best practices observed in existing studies (34–36). This consistency not only supports the robustness of our dataset but also enables comprehensive statistical analysis, ensuring that our findings are reliable and can be generalized to a broader population.

### 2.2 Measurement protocol

**Figure 1:**
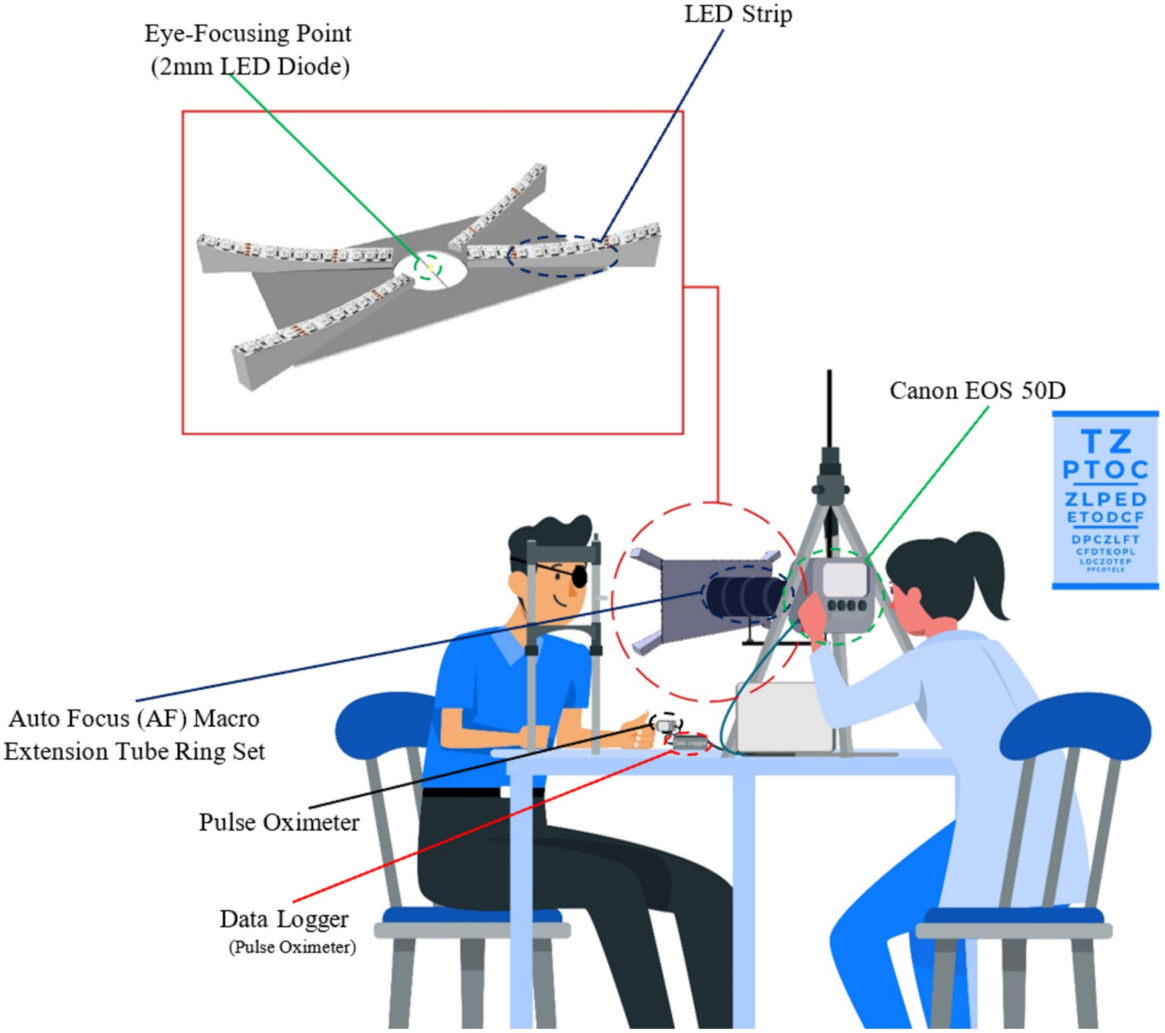
Measurement protocol overview.

A custom-designed prototype (Figure 1) with the Canon camera was used for the non-invasive recording of the reflecting LEDs illuminating the corneal surface, as shown in Figure 1 scheme. This custom-designed prototype offers enhanced resolution and accuracy over traditional videokeratometers, paving the way for more detailed insights into corneal deformations. To facilitate the data analysis process, 4 LEDs (1 LED in each fork, as shown in Figure 1) were considered, and the rest were covered. The primary diode, positioned near the pupil’s center, served as a focal point for eye fixation during measurements. The other four diodes, located at the corneal periphery, facilitated additional data capture. Video sequences were recorded from the right eyes of the subjects at the frequency of 25 fps. Synchronously, a numerical pulse oximeter acquired the blood pulsation signal with a frequency of 100 Hz. As head movements affect the measurement accuracy of the corneal deformations and light spot displacements, an ophthalmic headrest was applied to stabilize the head position. As was shown in the paper (33), the ophthalmic headrest stabilizes the head movements only partially, and quasi-periodical head movements due to blood pulsation transfer to some degree from the head based on the headrest. However, such quasi-fixation of the head significantly facilitates experiments and image analysis.

### 2.3 Data analysis

In order to synchronize recorded images of points reflected from the cornea and pulse oximeter signal, the start time of the video sequences coincided with the appropriate time of the blood pulsation signal. To make the spectral and coherence analysis for the same frequency, only every fourth sample of this blood pulsation signal was taken into account to get the same frequency 25 Hz as the frequency of recorded images. The video sequences were divided into frames in MATLAB software, and then the specific rectangular area corresponding to each test was considered, including the LED reflections. Each bright reflection spot from the cornea contained a dozen or several dozens of pixels. The center of mass of each bright spot was calculated to increase the accuracy of calculations of the corneal deformations and to get subpixel accuracy. Due to mass center calculations of each reflection from the cornea, the accuracy of measurements was significantly smaller than one pixel.

**Figure 2:**
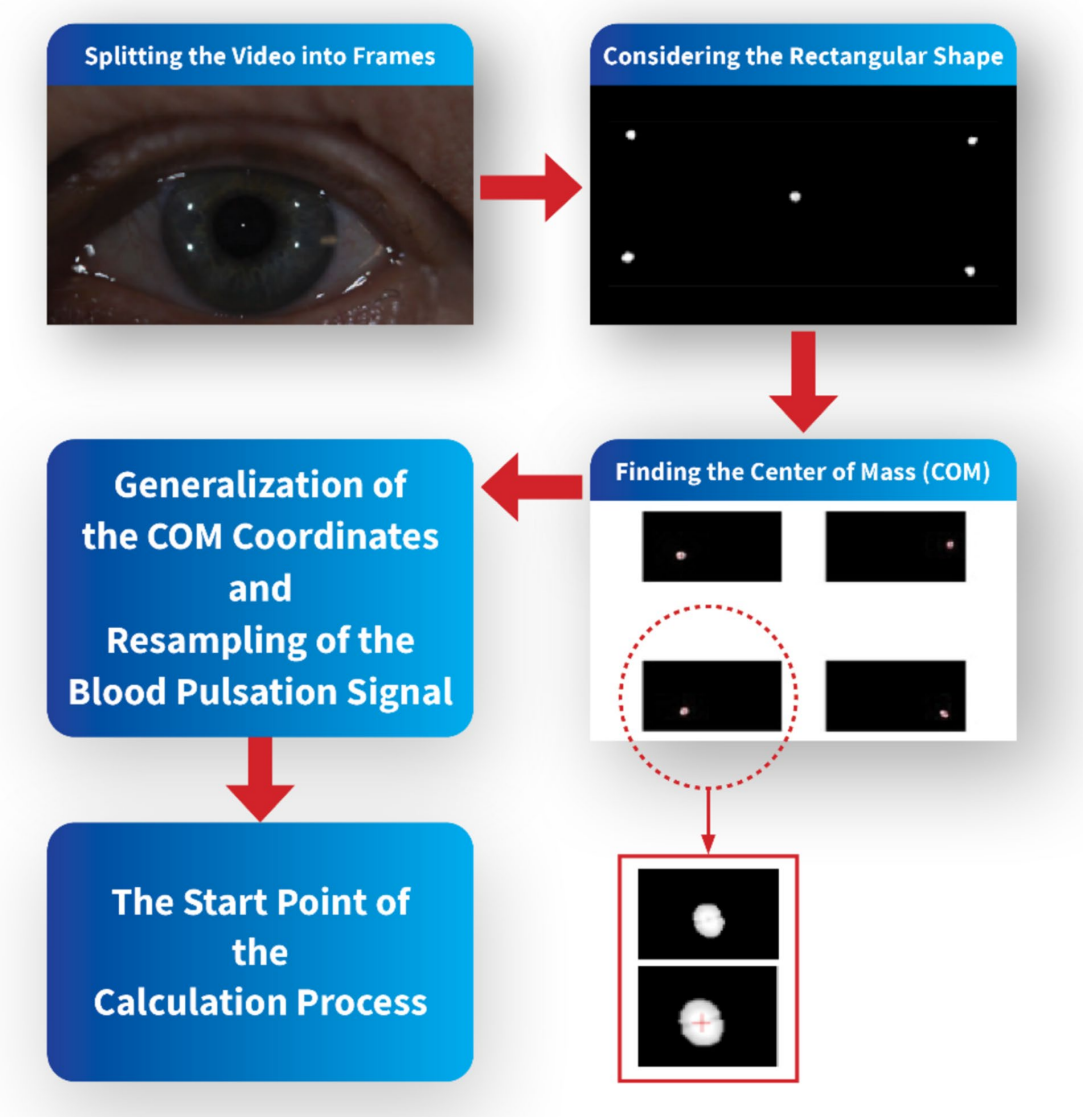
Schematic of the pre-calculation/processing flow.

The coordinates of these mass centers in each picture were next analyzed numerically to find variability of their positions and orientations of their movements. These data describe eye movements during measurements. Analysis of distances between appropriate spot mass centers shows deformations of the corneal surface. Figure 2 shows a simple schematic of the pre-calculation flow.

**Figure 3:**
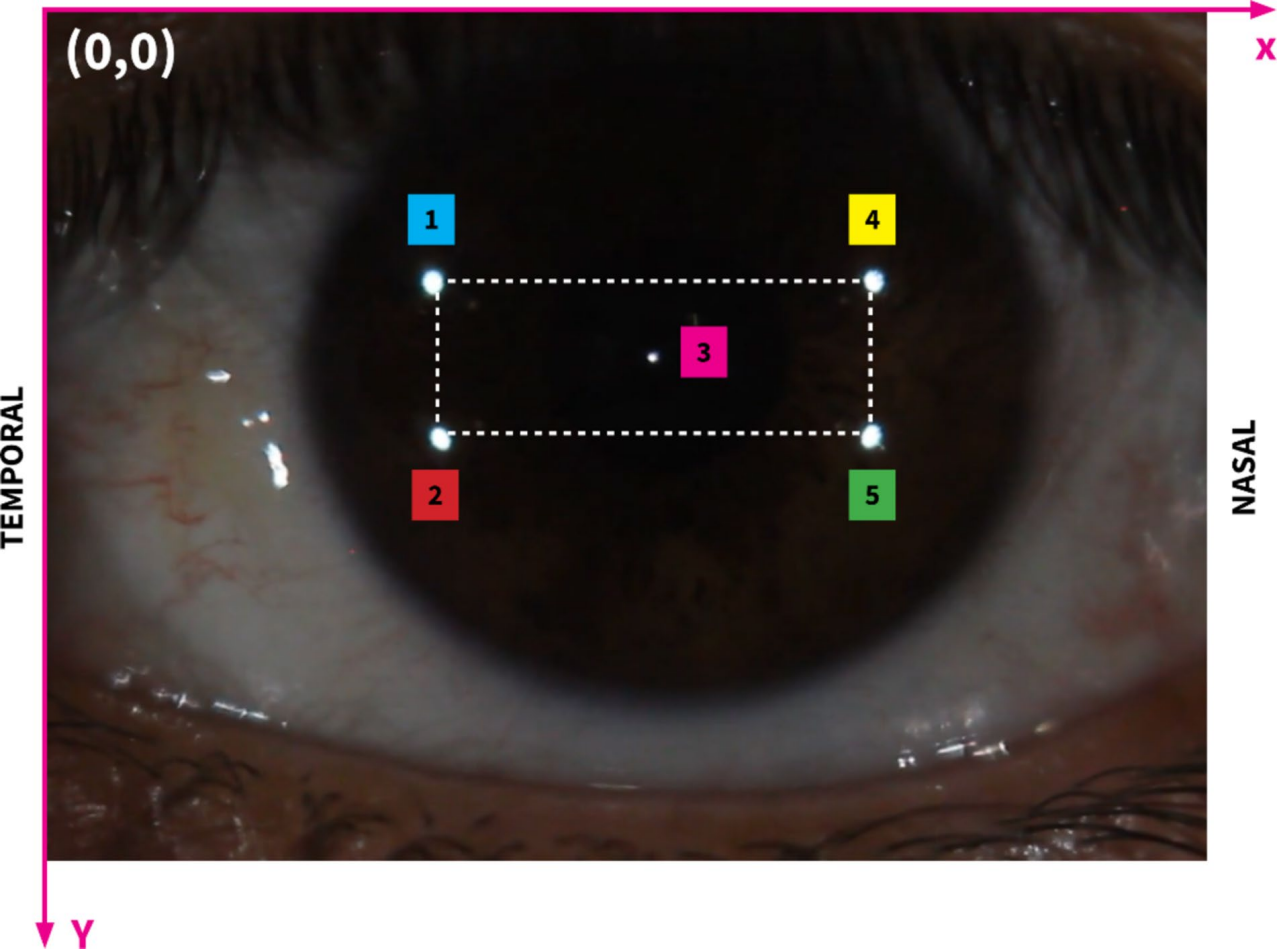
Image showing the region of interest on the corneal surface with the highlighted rectangular area. The rectangle encompasses the primary area of bright reflection spots that are critical for our analysis. This specific range facilitates a focused examination of the interactions between these spots and the corneal deformations. Subscripts 1 to 5 illustrate different bright reflection spots, their positions and distances.

Figure 3 presents five analyzed reflection points on the cornea located in the coordinate system and their numbers. To simplify the calculation process, the bright reflection spot will be identified with subscripts ranging from 1 to 5, as shown in Figure 3.

To precisely assess changes in corneal curvature, we focused on measuring the distance between specific reflective points on the corneal surface, with primary attention given to d24, the diagonal spanning from point 2 (temporal side) to point 4 (nasal side) on the cornea. This oblique distance, as illustrated in Figure 3, is the diagonal of the rectangular configuration formed by the reflective points. By positioning d24 along this diagonal, we capture the combined effects of horizontal (nasal-temporal) and vertical (superior–inferior) deformations, offering a more holistic measure of corneal curvature variations.

The rationale behind selecting d24 as a curvature indicator lies in its sensitivity to changes across multiple axes, which is particularly valuable when assessing corneal deformations. Corneal curvature directly influences the spatial relationships between fixed surface points: when the curvature steepens, the radius of curvature decreases, leading to a contraction along the diagonal, thereby reducing d24. Conversely, as the cornea flattens, the radius of curvature increases, and d24 expands. This geometric relationship allows d24 to reflect subtle adjustments in the corneal surface shape. The orientation of d24 as a diagonal distance makes it particularly responsive to variations that occur along both the horizontal and vertical planes, effectively capturing overall curvature changes rather than limiting the analysis to a single direction.

Additionally, this approach is consistent with established reflection-based measurement techniques in corneal topography and keratometry, where inter-point distances are commonly used to infer surface curvature. By tracking the shifts in d24, which represents the corneal surface’s response to internal and external forces, we gain insights into the biomechanical dynamics at play. The reliability of this measure is further enhanced by our use of high-resolution imaging and subpixel tracking, which ensure the precision and accuracy needed for such detailed curvature assessment.

**Figure 4:**
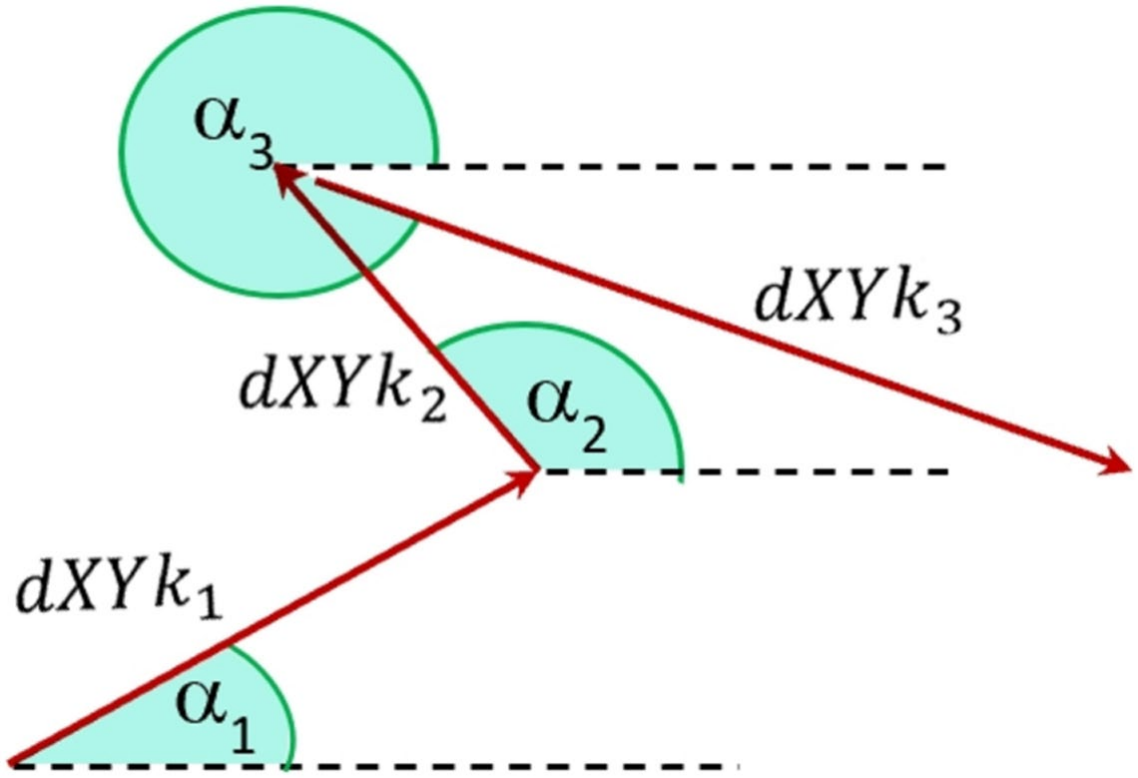
Schematic notation of orientation angles a [0–360 deg] of fast eye movements of the diode reflection on the cornea between three separate frames, where *k* represents the number of analyzed points.

Figure 4 provides a schematic representation of the orientation angle *α* of the analyzed reflection across three consecutive frames. The orientation angle α is utilized to examine directional variations in corneal curvature. Defined as the angle between the line connecting reflective points and the horizontal (nasal-temporal) axis, *α* allows us to assess how curvature changes differ across orientations. This parameter is essential for evaluating the anisotropic nature of corneal deformations and determining whether systemic influences, such as intraocular pressure and pulsatile ocular blood flow, affect corneal dynamics differently along specific axes. By exploring α, we aim to identify any directional dependencies in the cornea’s biomechanical response (37, 38).

Time and frequency domain analyses were conducted on the signals and their derivatives using MATLAB and MATHCAD software. Derivative-based analysis is crucial for capturing the rate of change in the signals, providing insights into the dynamics and rapid fluctuations that might not be apparent from the raw data alone.

Frequency domain analysis, though informative, often falls short in fully describing non-stationary signals whose characteristics change over time. To address this limitation, we utilized Short-Time Fourier Transform (STFT) with Hamming Windows. The STFT allows for a time-localized frequency analysis by segmenting the signal into overlapping windows, thereby enabling the observation of how spectral characteristics vary temporally. The application of the Hamming Window enhances the accuracy of the frequency domain representation by reducing spectral leakage and improving the clarity of the results. Additionally, the coherence function was employed to elucidate the spectral correlation between two signals within the frequency domain. Coherence measures the degree of correlation between the frequency components of the signals, providing valuable insights into the interrelationship between corneal deformations and ocular pulsations.

### 2.4 Validation of measurement method

To ensure the reliability and accuracy of our measurement method and to minimize the impact of potential confounding factors such as corneal surface roughness and tear film evaporation, we implemented specific participant selection criteria and standardized measurement protocols. Young healthy participants were carefully screened to exclude individuals with known ocular surface abnormalities, dry eye symptoms, or corneal conditions that could affect the quality of corneal reflections. Prior to each measurement session, participants were instructed to blink naturally to refresh the tear film, ensuring a smooth and uniform corneal surface. The measurement protocol required participants to refrain from blinking for approximately 10 s during data acquisition; however, if a participant blinked during this period, the measurement was paused and restarted to maintain data integrity. Repeated measurements were conducted for each participant to assess the consistency and repeatability of the data. The standard deviation of these repeated measurements was calculated to evaluate equipment variability, confirming that the fluctuations observed in the live data were significantly greater than the inherent variability of the measurement system. This approach ensured that our measurements reliably captured physiological changes in the cornea rather than artifacts arising from equipment limitations or external factors.

Moreover, in this study, all displacement and distance measurements of the corneal reflection points were recorded in pixel units. Due to the consistent experimental setup, camera settings, and equipment configuration across all measurements, we were able to compare relative changes in pixel units between participants and across different time points. This approach allowed us to analyze patterns and correlations within the data effectively.

## 3 Results

Exemplary results for one measurement of a young subject, AD, are provided in this chapter on behalf of the entire recorded data. The captured video series consists of *N* = 325 frames, and this sequence took 13 s to complete. The presented results display specific parameters that were primarily obtained for two selected images on the cornea, specifically point No. 2 and 4, in each measurement. Nonetheless, numerical analysis was performed for all five marked points. The achieved subpixel accuracy in determining the position of these points enabled a precise, comprehensive examination of both corneal deformations and eye movements. To evaluate the consistency of our findings across all subjects, we conducted a group-level statistical analysis. Correlation coefficients were calculated for each parameter pair: d24 (distance variability between points 2 and 4 on the corneal surface), dSC (variability in the quadrangle area formed by points 1, 2, 4, and 5), and dPu (derivative of the blood pulsation signal). The mean correlation coefficients were 0.66 (d24 vs. dPu), 0.59 (dSC vs. dPu), and 0.71 (d24 vs. dSC), with corresponding standard deviations of 0.05, 0.06, and 0.04, respectively. Each parameter pair showed statistical significance (*p* < 0.01), suggesting a reliable association across the cohort. These results indicate consistent relationships between corneal dynamics and cardiovascular activity among all eight subjects, supporting the generalizability of our observations within the context of this pilot study.

**Figure 5:**
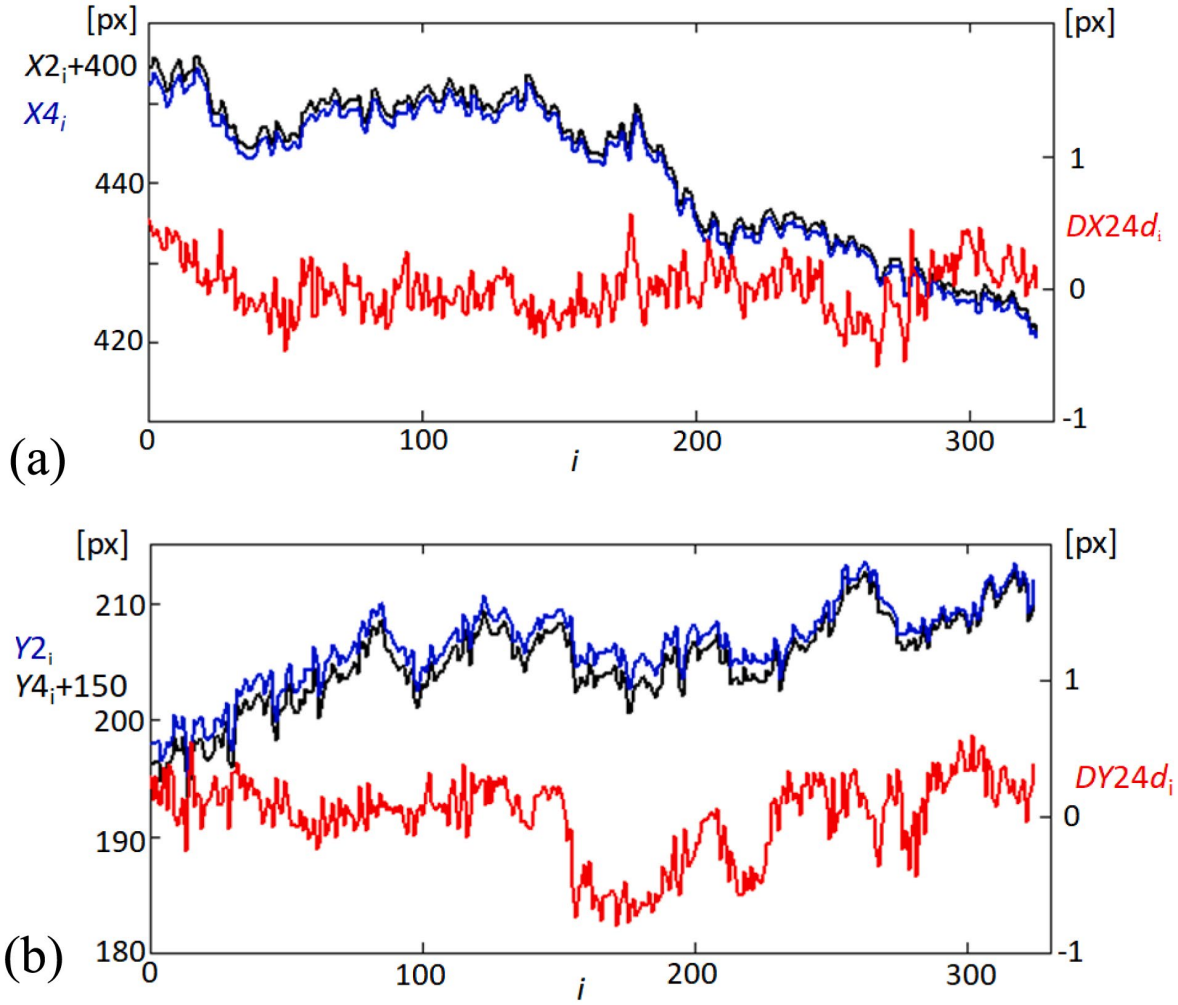
Temporal variability in corneal coordinates and their differences. **(A)** Graph showing the variability in the *X*-coordinates of points 2 (X2, black line) and 4 (X4, blue line) on the cornea, offset by adding 400 pixels to X2 for visualization clarity. The red line (DX24d) illustrates the difference between these coordinates, linearly detrended to highlight relative movements. **(B)** Corresponding graph for *Y*-coordinates, showing Y2 (blue line) and Y4 (black line), offset by adding 150 pixels to Y4. The red line (DY24d) depicts the detrended difference between these coordinates, emphasizing the variability over time.

Figure 5A illustrates the *X*-coordinates for point 2 (X2, adjusted by adding 400 pixels for clearer comparison, shown as the black line) and point 4 (X4, shown as the blue line). The red line (DX24d) represents the linearly detrended difference between these two points, highlighting the dynamic distance changes over time. Figure 5B displays the *Y*-coordinates for point 2 (Y2, blue line) and point 4 (Y4, adjusted by adding 150 pixels, shown as the black line). Similarly, the red line (DY24d) shows the detrended difference between the Y-coordinates of these points, providing insights into the vertical relative movement dynamics. Detrending is applied to the position data to remove any overarching linear trends, allowing for a clearer observation of the subtle, short-term variations in corneal dynamics. By isolating these fluctuations, we can better capture and analyze the directional deformations of the corneal surface. This process enhances the focus on biomechanical responses that may be related to influences, such as pulsatile ocular blood flow or fixational eye movements. This detailed tracking of corneal positions and their relative changes is crucial for understanding the biomechanics of eye movements in relation to the corneal structure.

**Figure 6:**
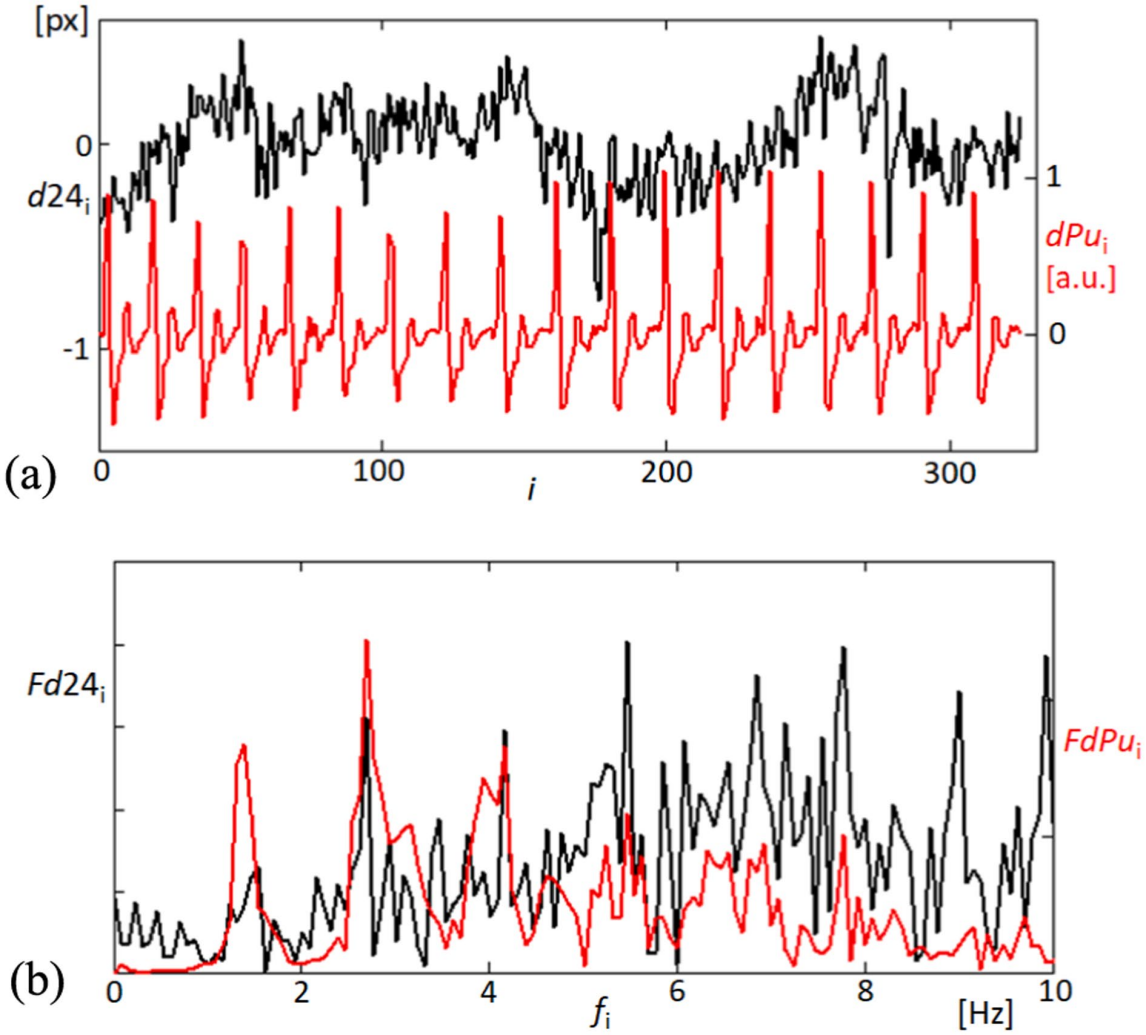
Temporal and frequency analysis of corneal displacement and blood pulsation signals. **(A)** The top panel illustrates the variability in the linearly detrended distance between points 2 and 4 on the cornea (d24, black line), alongside the derivative of the blood pulsation signal (dPu, red line), showcasing dynamic physiological interactions. **(B)** The bottom panel displays the Fourier spectra of both d24 (black line) and dPu (red line), revealing the frequency components up to 10 Hz.

In the exploration of corneal and cardiovascular interactions, Figure 6 provides a dual perspective on both temporal and spectral dynamics. Figure 6A captures the time-domain fluctuations of the linearly detrended distance between corneal points 2 and 4 (d24), place side by side with the derivative of the blood pulsation signal (dPu). The graphical representation highlights not only the magnitude of changes but also the temporal synchronicity between corneal motion and blood flow dynamics. Figure 6B extends the analysis into the frequency domain, where the Fourier spectra of d24 and dPu are plotted. The spectra elucidate the prevalent frequency components within each signal, with marked peaks indicating areas of significant physiological interaction. The correspondence of peaks across the two spectra suggests a strong harmonic relationship, potentially indicative of the biomechanical coupling between the cornea’s structural responses and blood pulsations. This comprehensive portrayal, through both temporal and frequency analyses, underscores the complex interdependencies characterizing corneal and cardiovascular behaviors. The observed spectral similarities affirm the hypothesis that corneal dynamics are closely linked with cardiovascular activities. The observed spectral similarities affirm the hypothesis that corneal dynamics are closely linked with cardiovascular activities, suggesting potential for non-invasive diagnostics that monitor these biomechanical interactions. To quantitatively assess the synchronization between the two signals, we applied cross-correlation analysis. This method calculates the similarity between the signals across time-lags, enabling us to identify the peak correlation coefficient and the time-lag at which synchronization is strongest. By demonstrating a high cross-correlation value near zero lag, we can quantitatively confirm the temporal alignment of the signals (39).

In our findings, significant correlations were observed between the variations in distances (d24) among corneal reflection points and the pulsatile ocular blood flow (POBF), suggesting synchronized corneal expansions with cardiovascular activity. The correlation between variations in d24 and POBF was primarily observed within the 1–8 Hz frequency range, a band associated with cardiovascular influences on ocular biomechanics. We applied coherence analysis to evaluate this relationship, focusing on this specific range. The high coherence values within this band indicate a significant correlation, capturing the dynamic interactions most relevant to POBF without suggesting a uniform correlation across the entire frequency spectrum.

**Figure 7:**
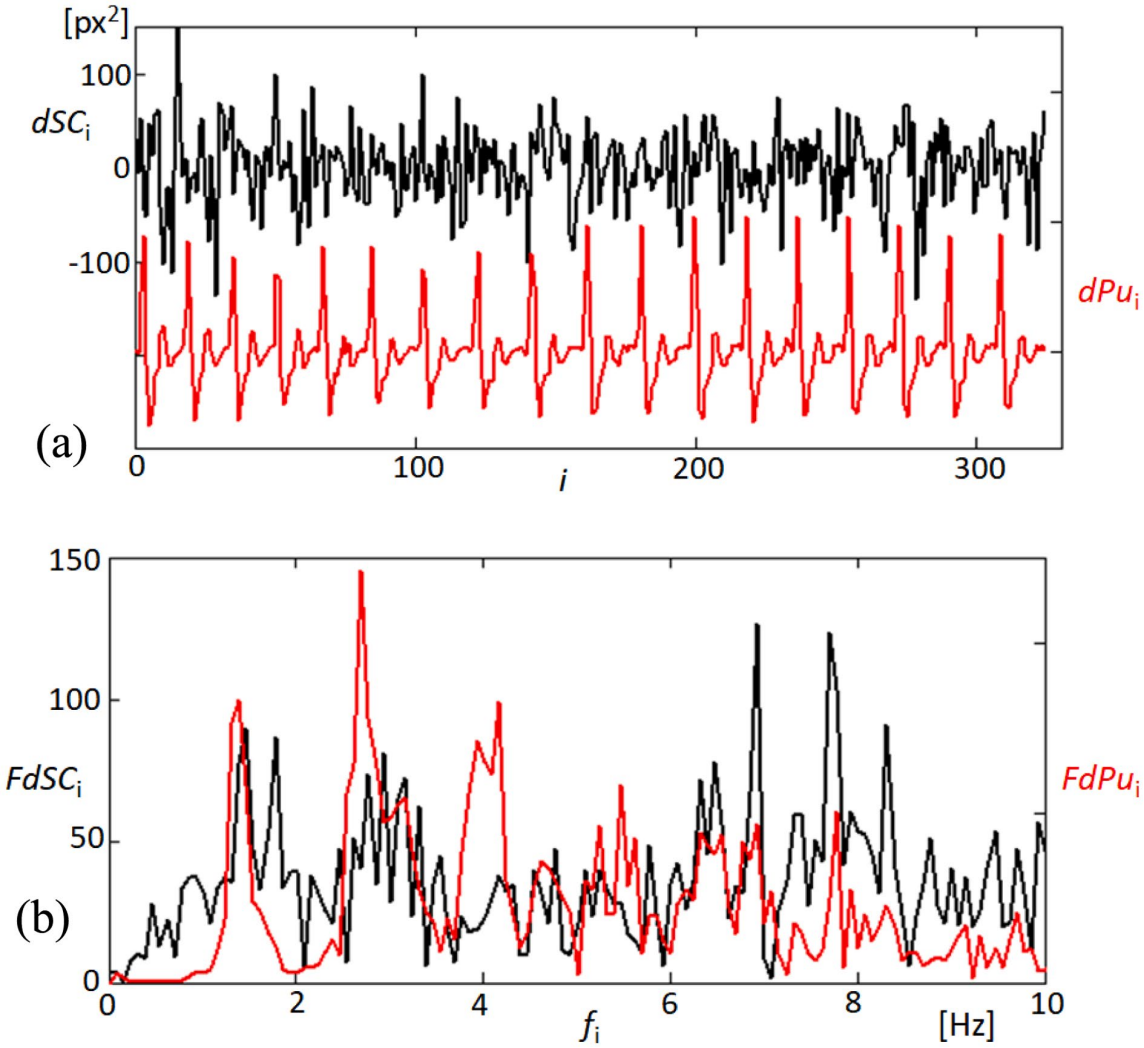
Analysis of quadrangle area dynamics and corresponding blood pulsation. **(A)** The top panel depicts changes in the quadrangle area (dSC, black line) formed by points 1, 2, 5, and 4 on the cornea, showing the linearly detrended variability in response to blood pulsation (dPu, red line). This graph reflects the corneal surface’s responsiveness to fluctuations in blood flow. **(B)** The bottom panel presents the Fourier spectra of both dSC (black line) and dPu (red line), illustrating the frequency components up to 10 Hz.

In examining the mechanical responsiveness of the corneal structure to cardiovascular dynamics, we specifically analyzed the variability of the area formed by points 1, 2, 5, and 4, designated as dSC. This area’s analysis, as shown in Figure 7A, was conducted to assess the homogeneous or heterogeneous nature of corneal expansion and contraction in relation to blood pulsation. The linear detrending applied to the area measurements highlights the cornea’s dynamic response to the rhythmic blood flow, as evidenced by the temporal correlation with the derivative of the blood pulsation signal (dPu). Further extending our analysis to the frequency domain, Figure 7B elucidates the spectral characteristics of both dSC and dPu. By examining the Fourier spectra, we observe significant peaks at specific frequencies, indicating potential frequencies where corneal deformation patterns align closely with cardiovascular signals. This spectral analysis not only confirms the temporal observations from the top panel but also enhances our understanding of the underlying physiological interactions, suggesting that certain frequencies of blood flow have a more marked impact on corneal dynamics. These findings contribute to a deeper understanding of the biomechanical properties of the cornea, particularly how it behaves in response to internal physiological changes. Such insights are crucial for developing predictive models of corneal behavior in clinical settings, potentially aiding in the non-invasive assessment of cardiovascular health based on ocular biometrics.

**Figure 8:**
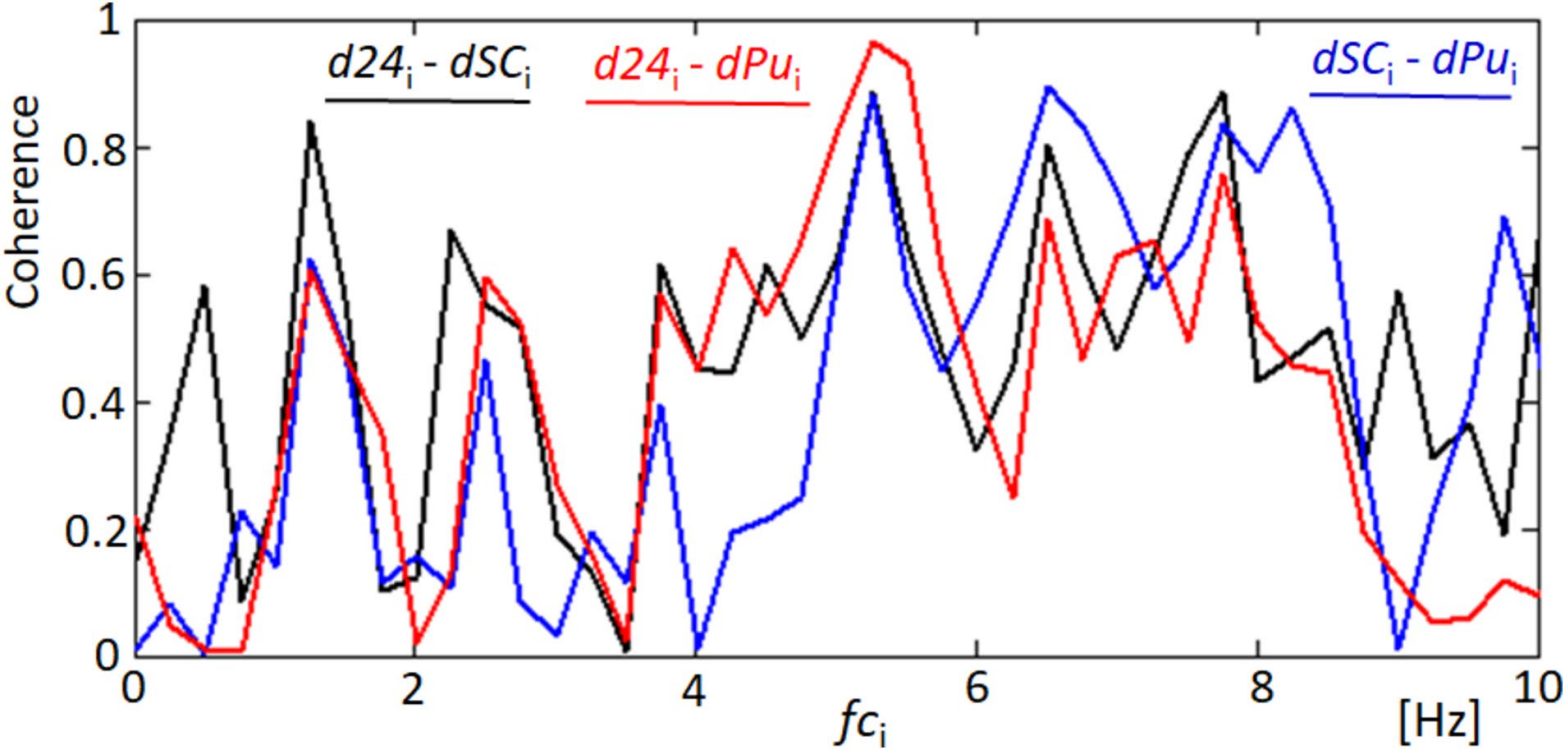
Coherence analysis between corneal and cardiovascular dynamics. This figure shows the coherence functions for three pairs of signals: d24 and dSC (black line), d24 and dPu (red line), and dSC and dPu (blue line). Significant coherence peaks are observed at key frequencies indicating synchronized responses that may correspond to blood harmonics.

Figure 8 focuses on the coherence analysis between pairs of signals derived from our measurements of corneal dynamics and cardiovascular activity. The coherence function, a statistical measure of the degree to which two signals are linearly related at each frequency, was utilized to assess the phase relationships between d24 (distance variability between points 2 and 4), dSC (variability in the quadrangle area), and dPu (derivative of the blood pulsation signal). The analysis revealed significant coherence at several key frequencies, particularly within the 5–8 Hz range. For example, the coherence between d24 and dSC, represented by the black line, shows notable peaks around 5 and 8 Hz, suggesting synchronized physiological responses between these two measures of corneal dynamics. Similarly, the coherence between d24 and dPu (red line) and between dSC and dPu (blue line) exhibits strong correlations at similar frequencies within the 5–8 Hz band. Although the cardiac pulse rate and its harmonics (e.g., 1.2, 2.4, and 3.6 Hz) are relevant, the significant coherence peaks occur at higher frequencies (5–8 Hz). This suggests that higher-order harmonics or other physiological factors may contribute to the observed synchronization. The high coherence values in this frequency range imply that the movements of the corneal surface and the variations in blood pulsation are not random but are indeed synchronized. The coherence at these specific frequencies suggests that corneal deformation and the cardiovascular system respond to the same physiological stimuli or share common regulatory mechanisms. Such findings underscore the complex interplay between ocular biomechanics and cardiovascular dynamics. These findings underscore the complex interplay between ocular biomechanics and cardiovascular dynamics, revealing phase relationships that could be critical for the development of non-invasive diagnostic and monitoring tools based on synchronized ocular signals.

**Figure 9:**
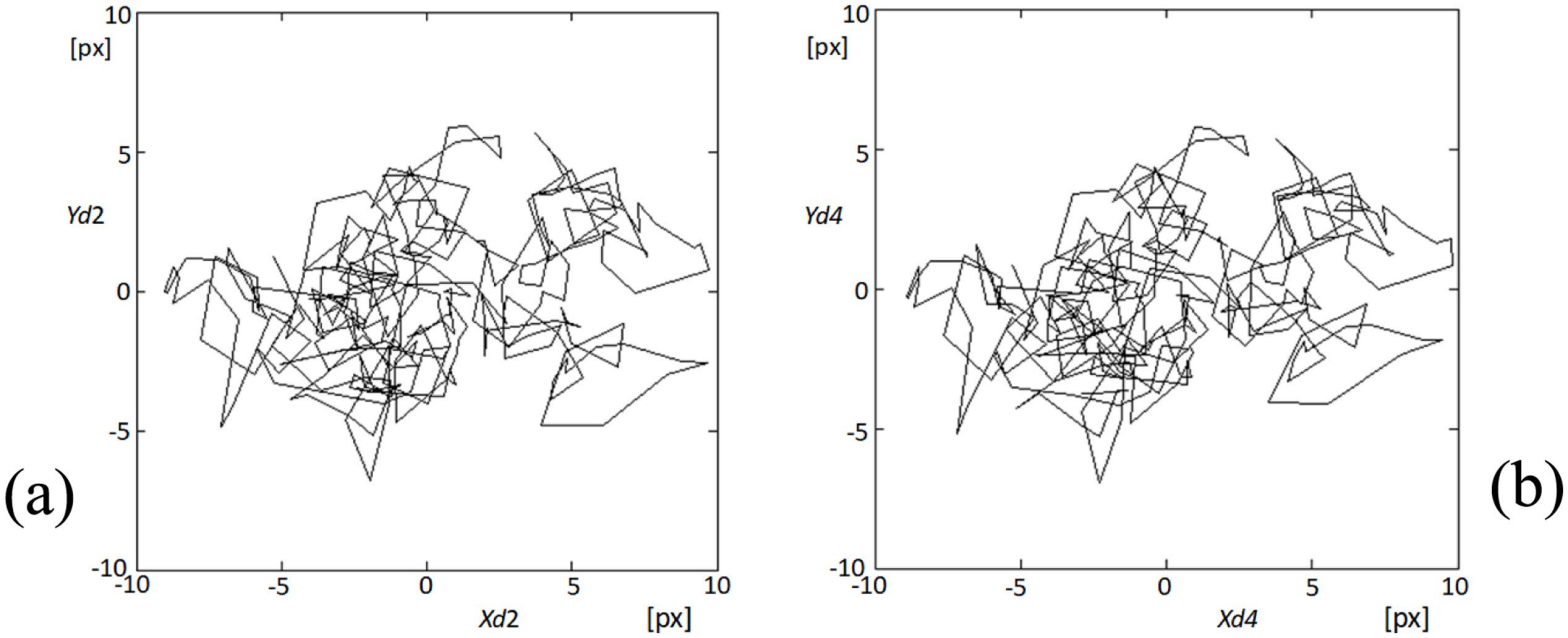
Visualization of fixational eye movements at points 2 and 4. **(A)** The plot shows the trajectories of fixational eye movements at point 2 (Xd2, Yd2) on the cornea, depicting complex movement patterns within the recorded video sequence. **(B)** Similarly, this plot displays the movement trajectories at point 4 (Xd4, Yd4), illustrating similar complex patterns to those observed at point 2, but with distinct, slight variations in trajectory dynamics.

In our analysis of fixational eye movements, detailed tracking of corneal points 2 and 4 provided insights into the dynamic behavior of these specific regions on the cornea. Figure 9 visually represents the trajectories of these points during the video sequence, captured as part of our comprehensive study on eye movement dynamics. Figure 9A depicts the trajectories at point 2, where complex patterns of movement are observable. Each line represents a separate trajectory over time, showing the variability and the extent of fixational movements within the observational window. Similarly, Figure 9B shows the movement patterns at point 4, which, while analogous to those at point 2, exhibit their unique characteristics and minor deviations in movement paths. The similarity in the patterns between these two points underscores the synchronized nature of fixational eye movements across different corneal regions. However, the subtle differences highlighted upon closer examination of the trajectories reveal the nuanced behaviors of each point, likely influenced by localized corneal biomechanics and broader ocular dynamics. These observations are pivotal in understanding the complexity of ocular fixational movements, which, despite their subtlety, play significant roles in visual stabilization and image processing. This analysis not only confirms the presence of these movements but also enhances our understanding of their behavioral patterns across the corneal surface.

**Figure 10:**
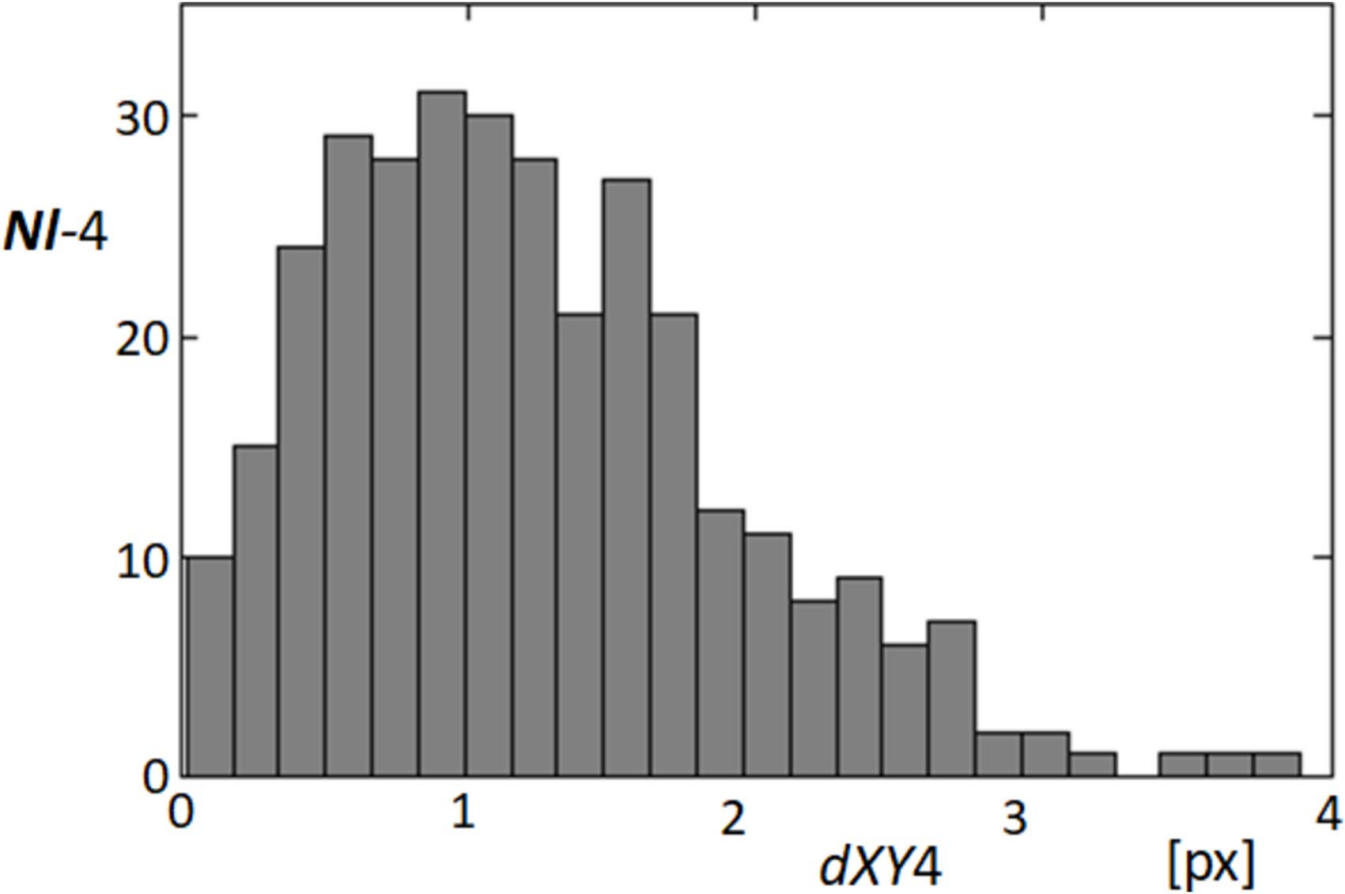
Histogram of fixational movement speeds at point 4. This histogram represents the distribution of speeds (measured in pixels per frame) for fixational movements at point 4, calculated from the distances moved from one frame to the next across the video sequence. The *x*-axis represents the speed in pixels per frame (dXY4), and the *y*-axis shows the frequency of these speeds occurring within the dataset.

In order to quantify the dynamic characteristics of fixational eye movements at point 4 on the cornea, we analyzed the distances traveled by this point from one video frame to the next over the entire sequence. Given a consistent frame interval of 40 milliseconds, these distances effectively represent the instantaneous speeds of the point’s movement. Figure 10 presents a histogram of these speeds, illustrating the distribution of movement lengths across 324 instances within the 325-frame sequence. The analysis revealed that the majority of these movements are relatively subtle, with the most common speeds being approximately 1 pixel per frame. However, the histogram also captures instances where speeds reach up to 4 pixels per frame, indicating occasional faster movements. This distribution provides insights into the nature of fixational eye movements, which are predominantly minor but can occasionally include more rapid shifts. Understanding these patterns is crucial for interpreting the stability and micro-movements of the eye, which have implications for both physiological studies and applications in visual tracking technologies.

**Figure 11:**
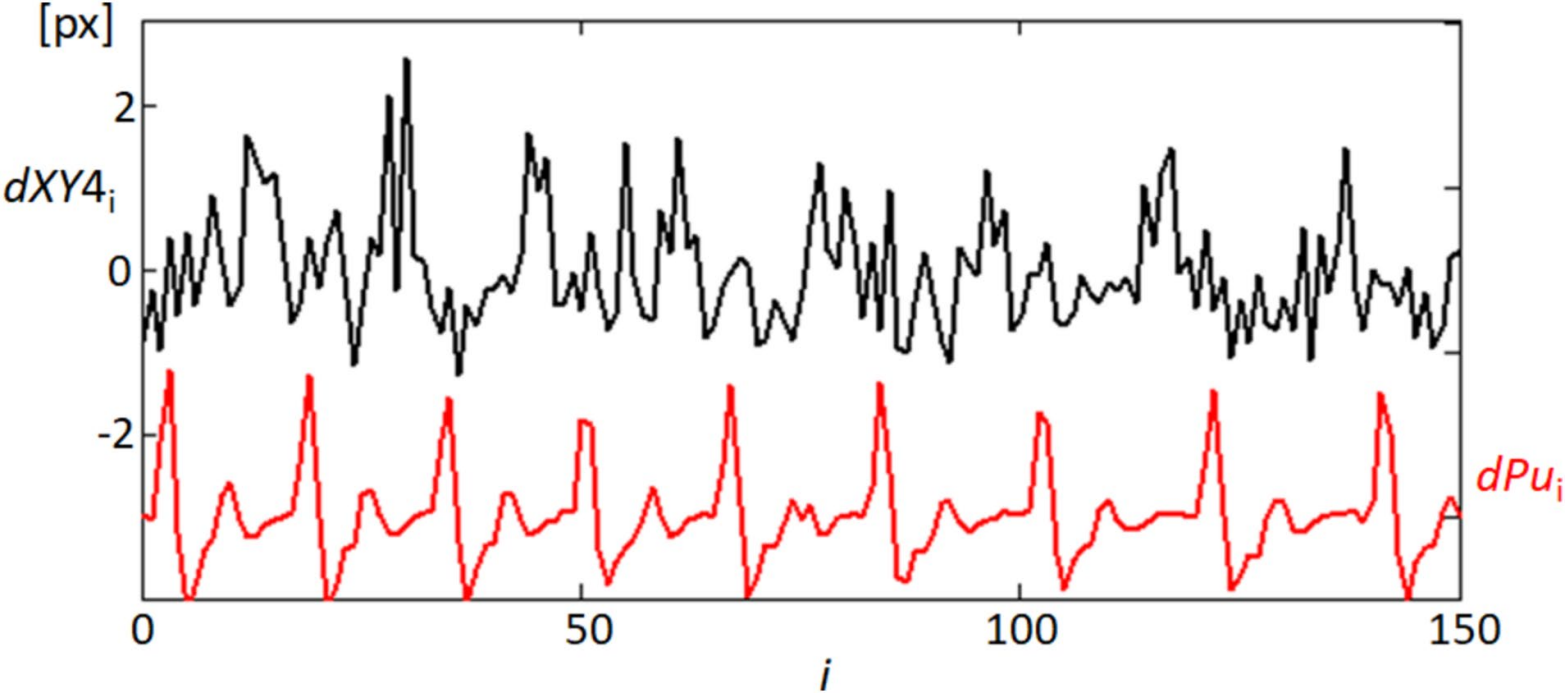
Temporal correlation of fixational eye movements and blood pulsation. This graph shows the temporal variability of movement lengths dXY4 (black line), representing the speeds of fixational eye movements at point 4, and the blood pulsation signal dPu (red line). Only the first half of the recording sequence is displayed to enhance clarity.

In our detailed examination of fixational eye movements, particularly focusing on the speeds of these movements as captured by the length of movement per frame (dXY4) at point 4, we observed notable temporal correlations with physiological signals. Figure 11 presents these findings, showing the variability in movement lengths alongside the derivative of the blood pulsation signal (dPu) over approximately half of the recorded sequence to ensure clearer visualization. The black line (dXY4) in the figure tracks the micro-movements, which are predominantly fixational eye movements such as microsaccades and drifts. These movements, while subtle, show a pattern of periodicity that interestingly aligns with the fluctuations observed in the red line representing the blood pulsation signal (dPu). This alignment may indicate that systemic physiological processes like blood flow could influence or synchronize with the subtle dynamics of ocular movements. While our analysis did not specifically differentiate between types of fixational movements, such as microsaccades or drifts, because the data was not detailed enough, the histogram of movement speeds suggests a relatively consistent distribution across the different types of movements. This pattern reinforces the understanding that fixational eye movements, even those of minimal amplitude, contribute significantly to overall ocular stability and may reflect underlying physiological rhythms. The correlation observed between these two measurements underscores potential areas for further research, particularly in the exploration of how health conditions might impact or be inferred from ocular movement patterns. The correlation between these two measurements opens up avenues for further research into how health conditions might influence or be inferred from ocular movement patterns, emphasizing the diagnostic potential of tools that integrate both ocular and cardiovascular dynamics.

**Figure 12:**
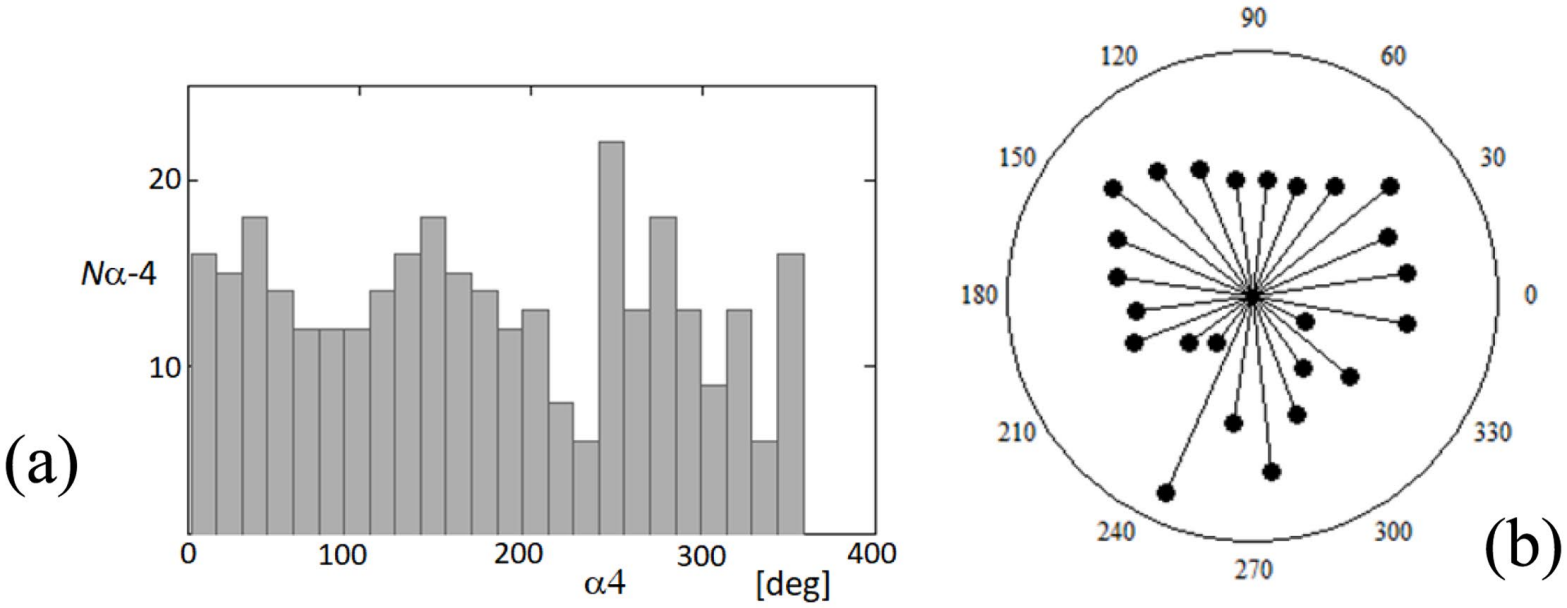
Distribution of orientation angles for fixational eye movements at point 4. **(A)** The histogram on the rectangular plot shows the distribution of orientation angles (*α*4) of fixational eye movements at point 4 over a complete 360-degree cycle. **(B)** The polar plot illustrates the same data, providing a visual representation of the directionality of these movements. Each point represents the frequency of movements at specific angles.

To further understand the dynamics of fixational eye movements, we analyzed the orientation angles of these movements at point 4 relative to the horizontal axis. Figure 12 captures the distribution of these orientation angles, which range from 0 to 360 degrees, representing the complete possible spectrum of movement directions. Figure 12A displays a histogram on a rectangular plot, categorizing the frequency of each orientation angle. This analysis shows a relatively uniform distribution across all angles, suggesting that there is no clear predominant direction in the fixational movements recorded. The movements appear isotropic, indicating that the eye may drift or make microsaccades in virtually any direction with similar likelihood. Figure 12B presents these data in a polar plot, which visually emphasizes the lack of a clear directional bias in these movements. The radial dispersion of points shows that while certain angles may have slightly higher occurrences, there is no significant concentration in any particular direction. This isotropic distribution is critical as it suggests that fixational eye movements, while seemingly random, provide the necessary minute adjustments for continuous visual perception and stabilization of the visual field. Understanding these patterns is essential for interpreting how the visual system maintains image stability despite constant small-scale movements. The lack of a predominant orientation further supports theories that fixational eye movements are adaptive responses to maintain retinal image variability, which is crucial for preventing sensory adaptation and enhancing perceptual sensitivity.

**Figure 13:**
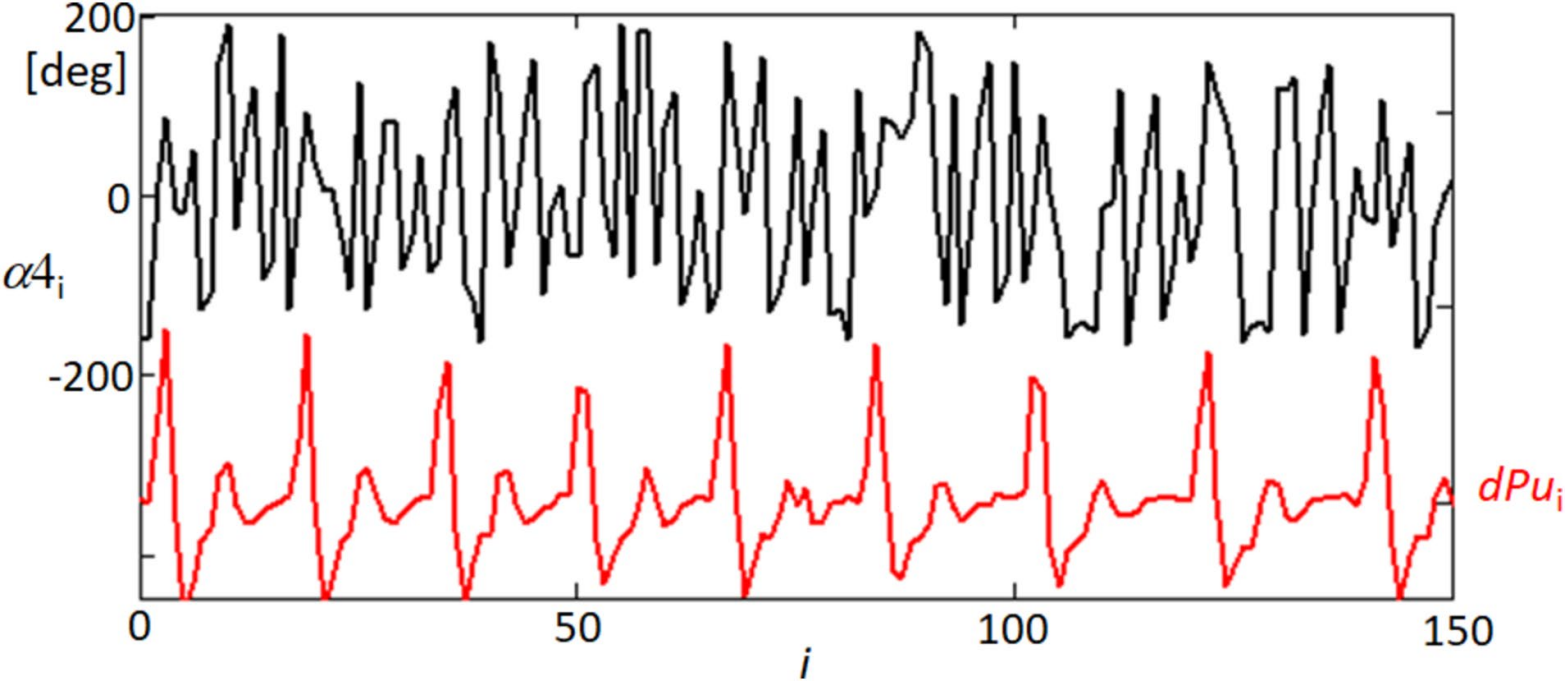
Temporal variability of orientation angle α4 and blood pulsation signal dPu. This graph displays the linearly detrended time variability of the orientation angle α of point 4 (α4, black line) alongside the derivative of the blood pulsation signal (dPu, red line) for approximately half of the recorded sequence. The detrending process adjusts α4 within a range of −180 to 180 degrees.

Figure 13 offers a detailed view of the temporal dynamics between ocular orientation and cardiovascular activity, specifically through the analysis of the orientation angle *α* at point 4 (α4) and its relationship with the blood pulsation signal (dPu). The angle α4 was processed through linear detrending to normalize its range between −180 and 180 degrees, facilitating a direct comparison with the rhythmic blood pulsation signals. The black line representing α4 in the figure illustrates the angle’s fluctuation over time, while the red line (dPu) indicates the derivative of the blood pulsation, capturing the instantaneous rate of change in blood flow. Observing these two signals together, the graph reveals periods where the variability in the angle and the pulsation signal appears synchronized, suggesting a potential interaction between ocular orientation dynamics and systemic blood flow. This synchronous behavior highlighted in the graph could reflect physiological interactions where vascular activities might influence or be reflected in the orientation dynamics of the eye.

**Figure 14:**
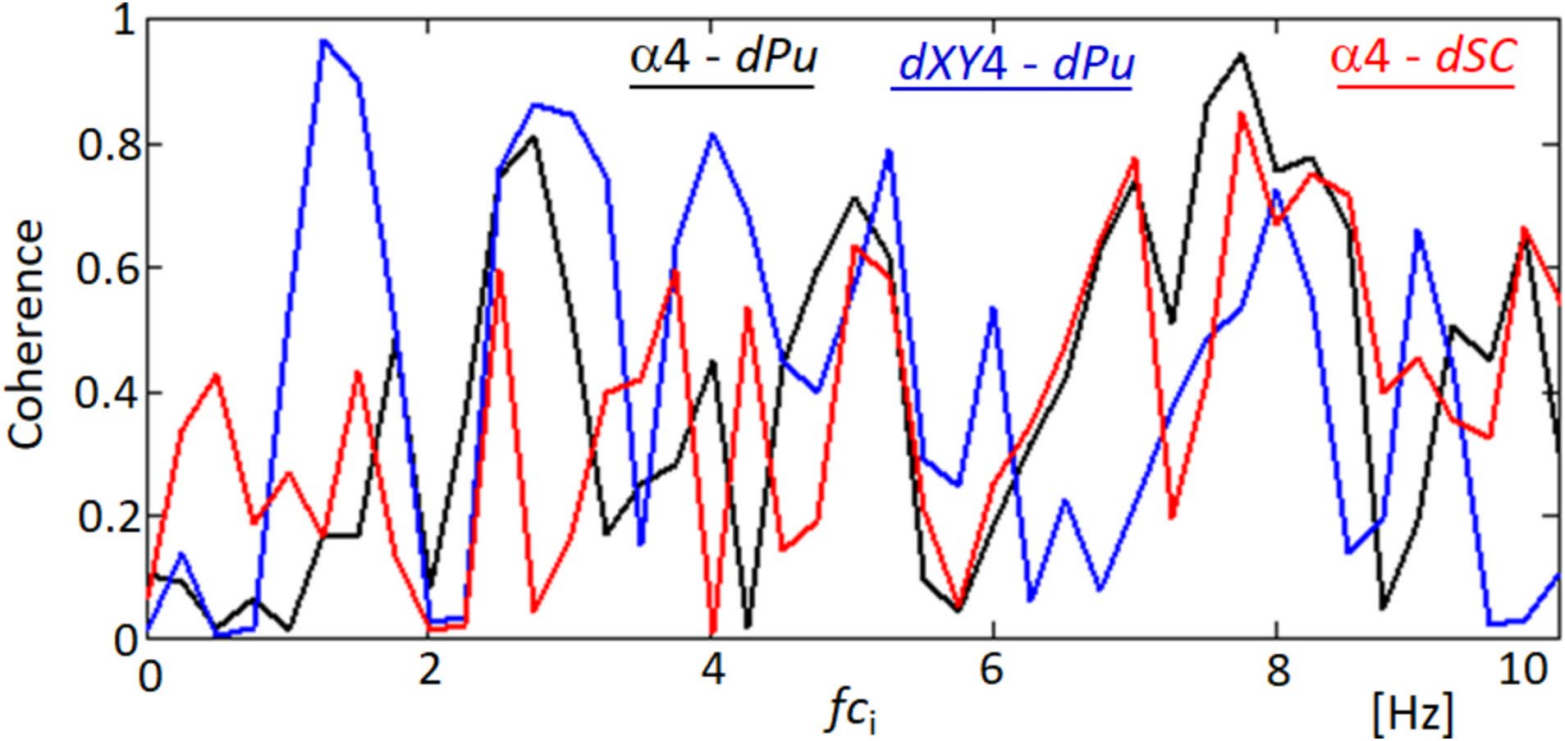
Coherence functions demonstrating correlations among ocular movement parameters. This figure shows the coherence functions between various parameters: orientation angle α4 and blood pulsation signal dPu (black line), lengths of movements dXY4 and blood pulsation dPu (blue line), and orientation angle α4 and the variability of the quadrangle area dSC (red line).

Figure 14 provides a spectral analysis of the coherence between different ocular and physiological parameters, revealing surprising correlations that underscore the complex interplay within the visual system. The coherence functions measure the degree of linear correlation between two signals at each frequency, and significant coherence suggests a possible physiological linkage or synchronized behavior between the variables being analyzed. The black line in the graph represents the coherence between the orientation angle α4 and the blood pulsation signal dPu, indicating periods of high correlation that could reflect the influence of cardiovascular dynamics on ocular orientation. Similarly, the red line illustrates the coherence between the lengths of movements dXY4 and the blood pulsation dPu, showing how the dynamics of eye movements are potentially synchronized with pulsatile blood flow changes. Most intriguingly, the blue line depicts the coherence between the orientation angle α4 and the variability of the quadrangle area dSC, suggesting that the orientation of eye movements may be related to broader changes in the corneal surface shape or tension, possibly driven by underlying physiological processes. These coherence results are significant as they provide a window into the multifaceted relationships that govern eye movements and their synchronization with physiological responses. Such findings could have implications for understanding how various factors, from blood flow to corneal biomechanics, interact to influence ocular stability and visual processing.

**Figure 15:**
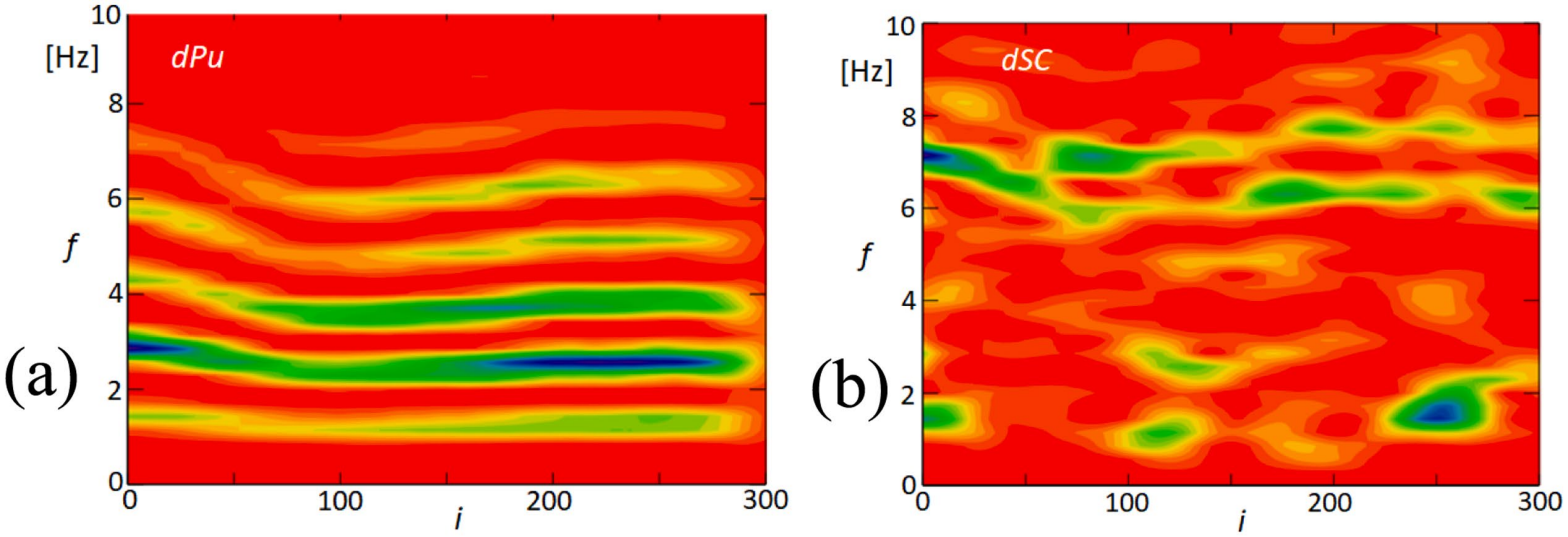
Time-frequency analysis of blood pulsation and quadrangle area variability. **(A)** This panel displays the time-frequency analysis of the blood pulsation derivative (dPu), highlighting the spectral components over time. **(B)** This panel shows the time-frequency analysis of the variability of the quadrangle area (dSC), illustrating how this parameter fluctuates across different frequencies. The color scale represents the amplitude of the signal, with red indicating high amplitude and blue indicating low amplitude.

Figure 15 offers a detailed exploration of the dynamic interactions between physiological signals and corneal surface behavior through time-frequency analysis. This approach enables us to observe fluctuations over time and how these variations are distributed across different frequency ranges. In Figure 15A, we focus on the derivative of the blood pulsation signal (dPu), illustrating how its intensity and frequency vary throughout the recorded sequence. The color gradients reflect consistent rhythmic components, which are particularly prominent in the lower frequency ranges. Figure 15B presents the variability of the quadrangle area (dSC), representing changes in the corneal surface. Both signals exhibit rhythmic components within the 2–6 Hz range, suggesting the presence of periodic behavior. While there is not a precise alignment in waveform patterns, the shared frequency range highlights similar rhythmic elements, contributing to our analysis of temporal patterns.

The similarity in these temporal frequency patterns between dPu and dSC aligns with the findings from our coherence analysis shown in Figure 8, which indicates consistent rhythmic elements within the 2–6 Hz range. This supports the hypothesis that cardiovascular dynamics may influence, or be reflected in, the biomechanical properties of the cornea.

Due to the constraints on the length of the article, the pertinent results regarding one measurement of the second subject, MI, have been included in Appendix 1 for detailed review. These results have been presented in a manner consistent with the format adopted in Figures 5–15, thereby maintaining continuity and ease of understanding. The recorded video sequence comprised 336 frames and spanned 13.44 s. Nearly all figures exhibit similar dependencies and correlations. However, the final two graphs in Supplementary Figures S10, S11 present some distinct dependencies. In place of a single coherence function between dXY4 – dPu, as shown in Figure 14, there is one coherence function between *α*4 – dXY4 in Supplementary Figure S10. Supplementary Figure S11B presents the time-frequency analysis of the orientation angle *α*, whereas Figure 15B displays the variability of the quadrangle area dSC.

## 4 Discussion

Our research aimed to investigate the corneal shape deformations that are correlated with quasi-periodic expansions of the ocular surface and displacements of the corneal apex, which are linked to heart activity. Previous studies (27, 40) have successfully observed and examined the axial displacements of the corneal apex and corneal surface deformations. However, these measurements primarily focused on linear displacements of different points on the cornea or sclera, typically measured in micrometers. It is important to note that corneal deformations should also manifest as changes in the distribution of corneal curvature. Therefore, it would be expected to observe these effects in a fine variation of size in videokeratoscopic images obtained from video sequences. However, previous examinations and analyses of such video sequences using videokeratometers did not yield clear results. One of the limitations was the small aperture of the camera lenses used in videokeratometers, which restricted the resolution and accuracy of the captured images. To overcome this limitation, the authors made the decision to employ a camera with a significantly higher aperture and perform a numerical analysis of the reflected corneal images with subpixel accuracy.

The analysis of the obtained results reveals specific variations in lengths such as d24 (distance between points 2 and 4) and d15 (distance between points 1 and 5, not included in the results section) and the area of quadrangle SC. These variations exhibit spectral correlations with the blood pulsation, as demonstrated by the coherence functions presented in Figure 8 and Supplementary Figure S4. The observed correlations suggest corresponding variations in corneal curvature, which also display a correlation with blood pulsation. The coherence analysis highlights strong correlations between specific frequency bands and physiological signals, particularly in the 1–3 Hz and 3–6 Hz ranges. The lower band (1–3 Hz) aligns with heart rate frequencies, reflecting the influence of pulsatile ocular blood flow (POBF) on corneal deformation. POBF, driven by the cardiac cycle, induces rhythmic intraocular pressure fluctuations, subtly impacting corneal shape. Meanwhile, the 3–6 Hz band likely reflects both cardiovascular-related microfluctuations and minor fixational eye movements, such as microsaccades, which influence corneal surface dynamics. This alignment supports the notion that cardiovascular rhythms and eye movement dynamics contribute meaningfully to corneal deformations within these frequency ranges, reinforcing our findings on the spectral coupling between the cornea and blood pulsation (27, 41, 42). Figure 15 illustrates that the time-frequency spectrum of dSC (variation in the distance of quadrangle SC) and the blood pulsation variations dPu exhibit similarities at frequencies around 5–8 Hz. Additionally, for other measurements, these similarities are observed at higher frequencies ranging from approximately 4–9 Hz, with varying magnitudes. This indicates that the variations in corneal curvature are spectrally correlated with blood pulsation. However, it is important to note that the accuracy of these measurements is lower than the accuracies achieved in papers (16, 43) where ultrasound heads measured displacements of the cornea and sclera. Variations in individual points’ *X* and *Y* positions, specifically points 2 and 4, are several times larger than in their mutual distance variations, d24. These relatively great variations of all five points’ *X*- and *Y*-coordinates represent FEM. The measurements of the five reflection points consistently exhibit very similar variations, as depicted in Figure 9 and Supplementary Figure S5. The presence of such similarities motivated us to conduct a thorough analysis of the selected properties of these variations and their potential correlations with blood pulsation, which is a reflection of heart activity. However, while these findings are crucial, it’s essential to consider other factors that might influence them. While our study provides novel insights into the relationship between FEM, corneal deformations, and heart activity, it’s worth noting the potential influence of head movements on FEM measurements. Although head movements were not independently measured in our study, their potential interference, especially during rapid movements, cannot be ignored. Future studies aiming for a more comprehensive understanding might benefit from integrated measurements that account for both FEM and fine head movements.

The interesting findings of our study include the quasiperiodic characteristics of the FEM speeds, representing the displacements of analyzed points between separate frames (within 40 ms), as demonstrated in Figure 11 and Supplementary Figure S7. Moreover, a notable high spectral correlation was observed between these FEM speeds and the blood pulsation, illustrated by the coherence function between dXY4 and dPu in Figure 14. In Figure 14, the coherence analysis reveals that the highest coherence values occur at approximately 1.3 Hz and 2.6 Hz (blue graph). These frequencies correspond to the blood pulsation’s first and second harmonics. Similarly, for the second subject (Appendix 1), the highest coherence values between the same signals appear at approximately 2 and 4 Hz, which are also the first and the second harmonics of the blood pulsation, as evident in Supplementary Figure S2. The results obtained in our study can be treated as an extension or generalization of the findings presented by Peng et al. (29). Specifically, our results demonstrate the spectral coupling between the FEM and the blood pulsation (heartbeat). Notably, this coupling takes into account Heart Rate Variability (HRV), as indicated by the high coherence values observed between dXY4 and Pu (blood pulsation) across a relatively wide range of frequencies, as depicted in Figure 14.

Figure 12 indicates that the orientations of the FEM (regardless of their speeds) are relatively independent of specific directions. In contrast, in Supplementary Figure S8, the orientations are clearly defined, with approximately 60- and 220-degree angles. However, experimental observations have shown that such distinct orientations of FEM movements can vary for the same subject. In cases where a measured subject exhibits a consistent orientation across multiple measurements, the specific directions tend to be relatively similar. The most unexpected results pertained to the temporal variations of movement orientations, specifically the angle *αk*, where *k* represents the image point number. The variations in α4 orientations, as illustrated in Figure 13 and Supplementary Figure S9, exhibit a distinct quasi-periodic character. Further analysis revealed their relatively strong spectral correlations, including the Fourier spectrum (not shown in the paper) and coherence functions with the blood pulsation and movement speeds (Figure 14 and Supplementary Figure S10). Notably, most investigations regarding FEM properties have focused on their relationships with visual processes and their role in foveal perception (32, 44, 45). However, our research brings novel insights by highlighting the intriguing temporal variations and spectral correlations associated with movement orientations, expanding the understanding of FEM dynamics beyond their visual implications. The remarkable resemblance observed between the values and variations of the second, third, and partially fourth harmonics of the blood pulsation (heart activity) and the α4 angles, as illustrated in Supplementary Figure S11, indicates a strong spectral correlation between these variables. Notably, the distinct waving effect of these three harmonics, with a period of approximately 3.5 s (i @ 90 frames), is likely associated with the subject’s breathing pattern.

One limitation of this study is the relatively small sample size of eight subjects. To ensure statistical robustness, we conducted a preliminary power analysis using G*Power software. This analysis, based on an anticipated effect size of *d* = 0.5, an alpha level of 0.05, and a power of 0.80, confirmed that eight subjects would provide a statistical power of 82% to detect significant effects, particularly in identifying changes in corneal deformations linked to cardiovascular dynamics. This approach aligns with similar exploratory studies (21, 38), which have also utilized small cohorts to investigate complex physiological interactions. While the high-resolution data collected enabled detailed analyses, larger studies are necessary to enhance the generalizability and validation of our findings. Another potential limitation of our study is the influence of confounding factors such as corneal surface roughness and tear film dynamics on the measurement of corneal deformations. Variations in the corneal surface or instability of the tear film could potentially affect the reflections of the LEDs and, consequently, the accuracy of our measurements. We mitigated these factors by implementing stringent participant selection criteria to exclude individuals with known corneal abnormalities or dry eye symptoms. Additionally, we standardized the measurement protocol by instructing participants to blink immediately before data acquisition and limiting measurement durations to approximately 10 s to minimize tear film evaporation. Measurements affected by blinking were repeated to ensure consistency and data integrity. We assessed equipment variability through repeated measurements under identical conditions, demonstrating that the observed fluctuations in corneal reflections were significantly greater than the system’s inherent variability. Moreover, the study lacks a calibration procedure to convert pixel measurements into physical distances. While the consistent experimental conditions and equipment settings allowed us to compare relative changes across participants effectively, we recognize that quantifying these changes in physical units would enhance the scientific significance of the results. Without calibration, it’s difficult to determine the exact magnitude of corneal deformations and assess their clinical relevance. In future studies, we plan to incorporate a detailed calibration process using a micrometric calibration grid or a model eye. This will allow us to establish the precise conversion factor between pixels and micrometers, enabling a more accurate interpretation of the corneal deformations and their physiological implications. By converting the measurements into physical units, we will be able to compare our findings with established biomechanical properties of the cornea and better understand the potential impact of these deformations on ocular health and function. This enhancement will also facilitate the comparison of our results with other studies and contribute to the advancement of knowledge in the field of corneal biomechanics.

Future advancements in imaging technology could allow for more precise three-dimensional assessments of the cornea. Techniques such as advanced corneal topography and three-dimensional biomechanical modeling could revolutionize our understanding of the corneal response to physiological stimuli, providing a more detailed exploration of its biomechanical properties.

The use of a standardized headrest significantly reduced the impact of head movements on our measurements, but achieving perfect immobilization remains challenging. Future research could focus on integrating real-time motion correction technologies or more sophisticated head stabilization systems that adapt to and compensate for subtle movements. Employing advanced data analysis techniques to account for movement artifacts could further enhance the accuracy and reliability of corneal movement measurements.

The temporal orientations of head movements and fixational eye movements (FEM) differ significantly due to the substantial difference in mass between the head and the eye, with FEM being faster and more complex. The influence of fast head movements on FEM remains an open question, as the effect of head movements is often overlooked and not measured in many FEM experiments. However, it is essential to recognize that head movements can significantly impact results and their interpretation. Fine measurements of head movements, including rotations and displacements, pose significant challenges. Achieving the desired accuracy in capturing head movements is crucial for high precision in FEM analysis. One approach is to mount a device on the head to record FEM and minimize head movements. However, this method has limitations, as contact with the head skin introduces potential confounding factors like blood pulsation, which can cause deviations from actual head movements. Developing techniques that accurately measure fine head movements without interference from external factors, such as blood pulsation, is crucial for ensuring the integrity and validity of FEM studies, ultimately enhancing our understanding of the relationship between head movements and FEM dynamics.

To our knowledge, the literature lacks previous presentations of similar findings. While Ohl et al.’s study demonstrates intriguing results primarily focused on microsaccades, it does not consider the influence of heart rate variability (HRV). This raises compelling questions about the potential impact or interdependence of the examined effects and observed correlations on visual processes. These influences could be associated with factors such as visibility during fixation or dynamic image quality, specifically on the fovea. Further research into adaptive optics effects, known to enhance image quality, and their relationship with fine and fast eye and head movements would be extremely interesting.

While our current study is observational and demonstrates significant correlations between cardiovascular activity and corneal biomechanics, we recognize the importance of establishing causality to fully understand the underlying mechanisms. Future research will focus on experimental designs that allow for controlled manipulation of cardiovascular parameters and the use of sophisticated analytical methods to analyze the resulting data. Moreover, we acknowledge that respiratory patterns could influence corneal dynamics and that simultaneous recording of breathing could provide valuable insights into this relationship. While our primary focus in this study was on cardiovascular-induced corneal deformations, future research will incorporate respiratory monitoring to explore its potential impact on the corneal surface. In addition to our core findings, it is essential to consider the broader physiological interactions that may influence our results. Specifically, fluctuations in cardiac activity are known to impact intraocular pressure (IOP), which can subsequently alter corneal deformation. Variations in blood flow and heart rate have been hypothesized to lead to corresponding changes in IOP, potentially affecting the biomechanical behavior of the cornea (34). These dynamics are crucial for understanding ocular biomechanics, particularly how health impacts eye function. Future studies should aim to integrate more comprehensive physiological monitoring to quantify these interactions more effectively, employing technologies that can simultaneously track cardiovascular dynamics and ocular biomechanical responses.

## 5 Conclusion

Our study provides compelling evidence of the complex interplay between corneal deformations, fixational eye movements, and cardiovascular activity. Through detailed biomechanical analyses, we demonstrated that variations in corneal curvature, particularly observed through distances such as d24 and d15, are significantly correlated with pulsatile ocular blood flow. These findings not only enhance our understanding of ocular biomechanics under cardiovascular influences but also offer potential avenues for developing non-invasive diagnostic methods that could leverage these dynamics.

Moreover, our research has opened pathways for exploring how these biomechanical insights could be applied in clinical settings, particularly in diagnosing and monitoring conditions that affect both ocular and cardiovascular systems. However, while promising, our conclusions are tempered by the recognition that our study’s limited sample size may constrain the generalizability of these findings. Therefore, future research with larger cohorts is crucial to validate and potentially expand upon our conclusions.

Finally, the potential influence of head movements on FEM measurements, while not directly measured in this study, highlights an area for further research. Addressing this challenge is essential for refining the accuracy of our biomechanical models and enhancing the applicability of our findings in real-world clinical and diagnostic settings.

## Data Availability

The original contributions presented in the study are included in the article/Supplementary material, further inquiries can be directed to the corresponding author.
