# Supplementary material for: Pilot study on the dynamic interactions between cardiac activity and corneal biomechanics during eye movements
Source: Front Med (Lausanne). 2024 Dec 10;11:1484449. doi: 10.3389/fmed.2024.1484449 (PMC11666380; doi:10.3389/fmed.2024.1484449)
Supplement: Supplementary file 1 [file Data_Sheet_1.PDF]

Supplementary Material

Appendix 1. Additional Results

The Appendix provides a comprehensive examination of the data concerning the second subject MI, allowing readers to explore the findings more deeply beyond what is detailed in the article's main text.

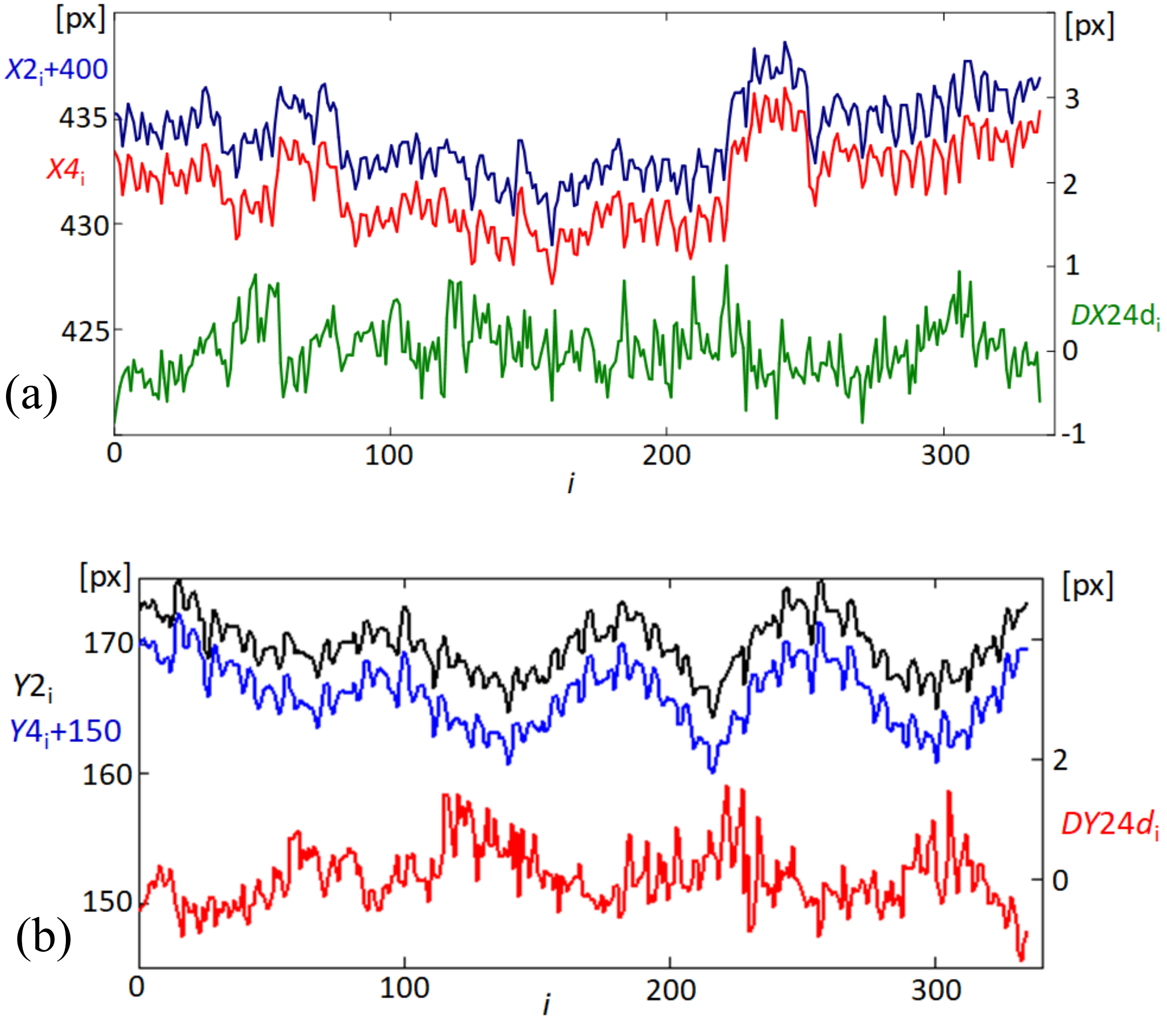

Figure 16. a) Variability of the X-coordinates of points 2 and 4 and their differences DX24d for all frames i, recorded in the video sequence, b) Y-coordinates of points No. 2 and No. 4 change in relation to frame number i and their differences DY24d. The graph shares similarities with Figure 5.

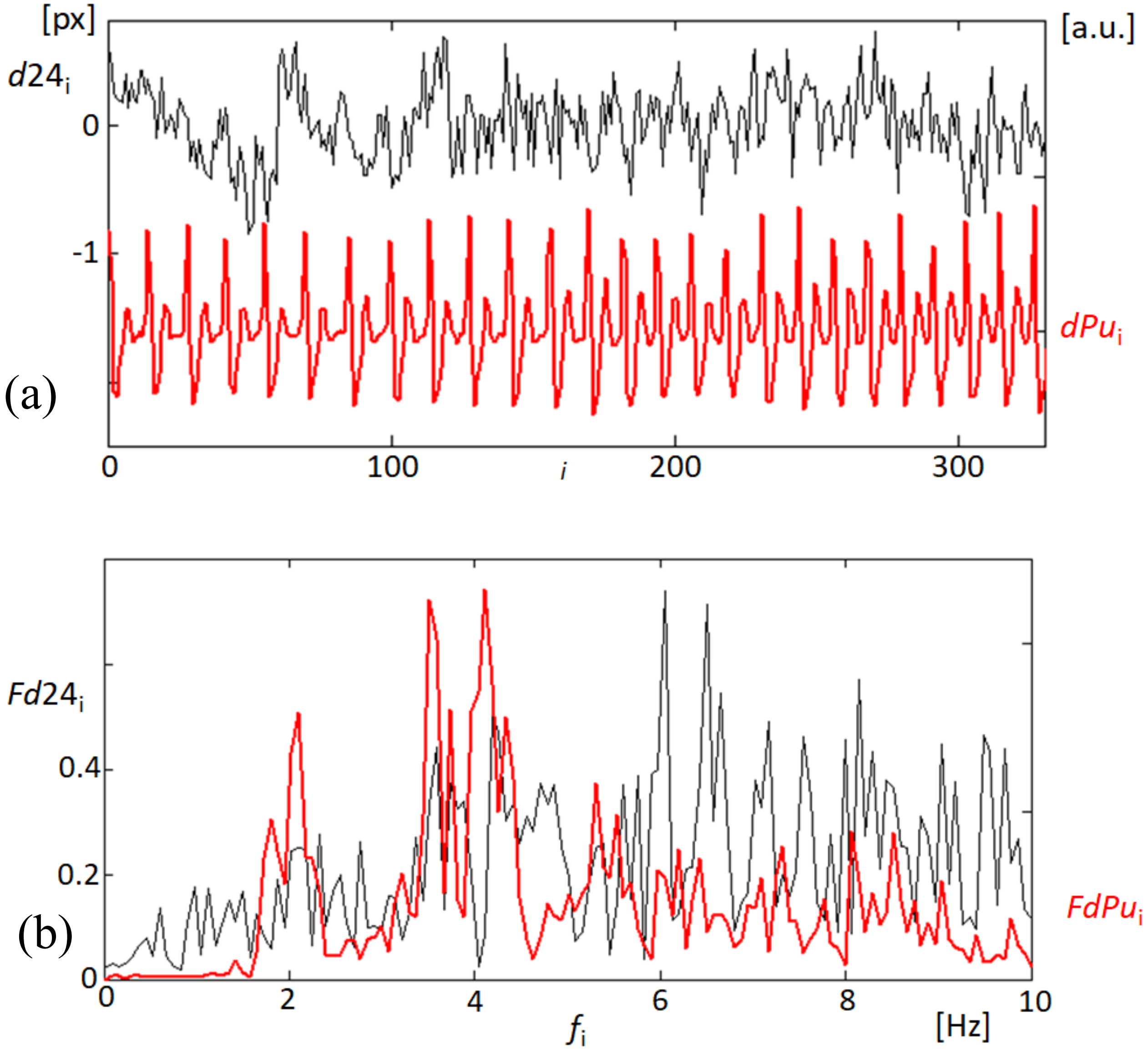

Figure 17. The distance variability between points 2 and 4 after applying a linear detrend, along with the corresponding derivative of the recorded blood pulsation signal. b) The Fourier spectrum of both signals, showcasing the frequency components present in each signal. The results are comparable to Figure 6.

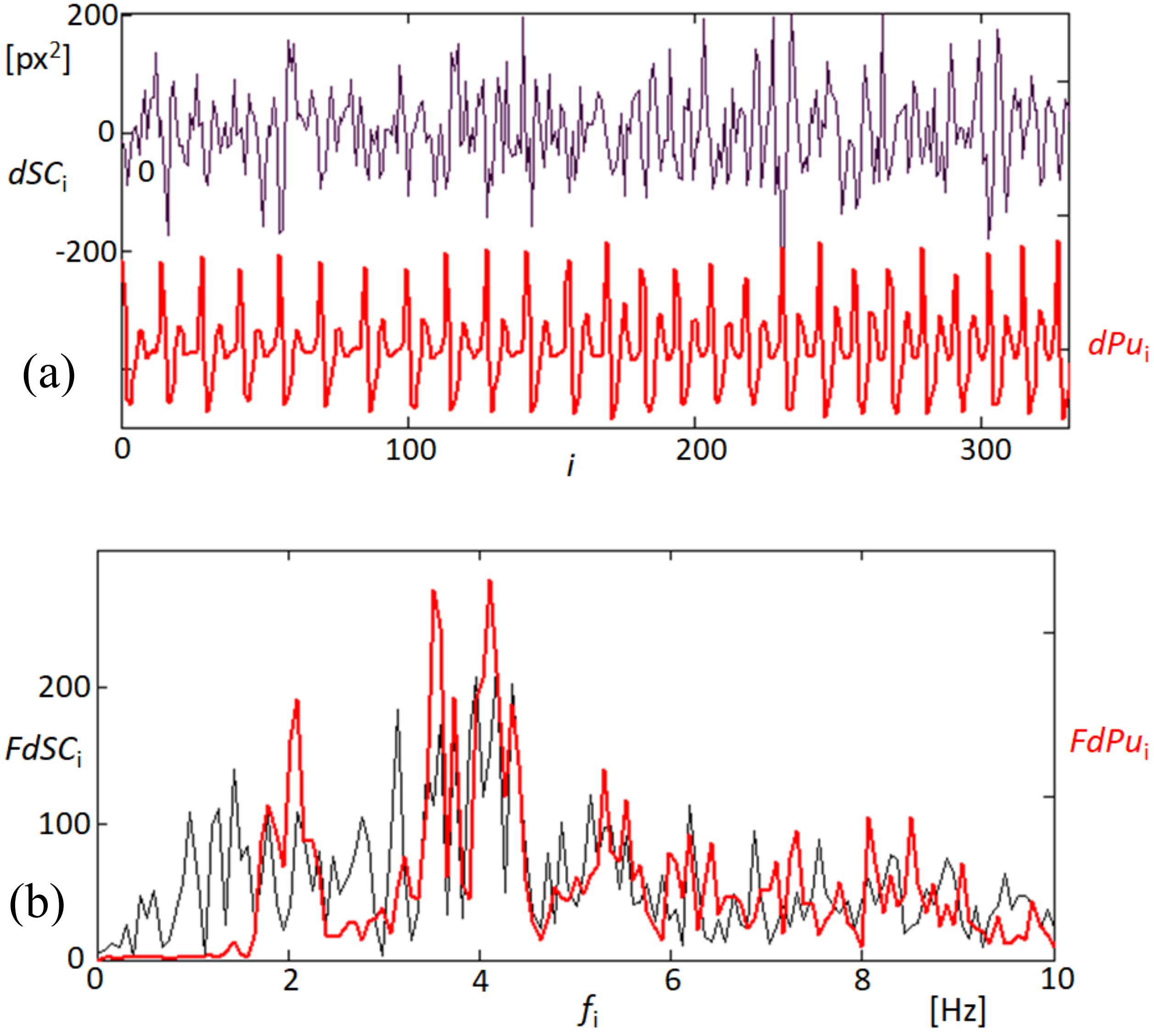

Figure 18. a) The variability of the quadrangle area, dSC, between points 1254 after applying a linear detrend, along with the recorded blood pulsation signal. b) The respective Fourier spectra of both signals, depicting the frequency components present in each signal. The graph shows a similar trend as Figure 7.

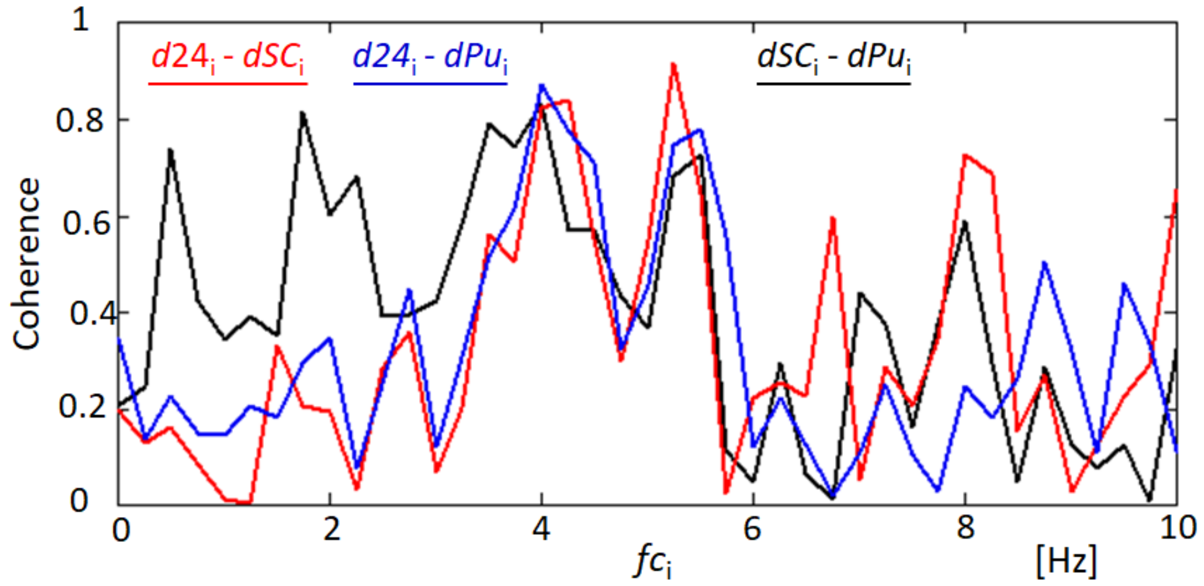

Figure 19. Coherence function between three pairs of respective three signals. The graph exhibits similarities to Figure 8.

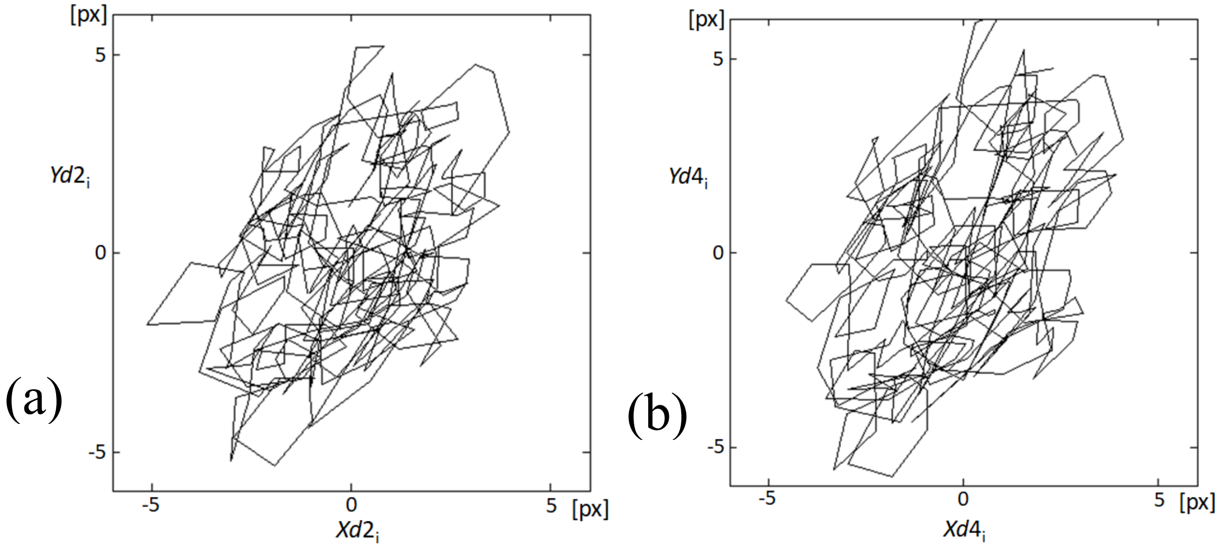

Figure 20. Fixational eye movements during recorded video sequence a) point 2, b) point 4. Consistent with the findings depicted in Figure 9.

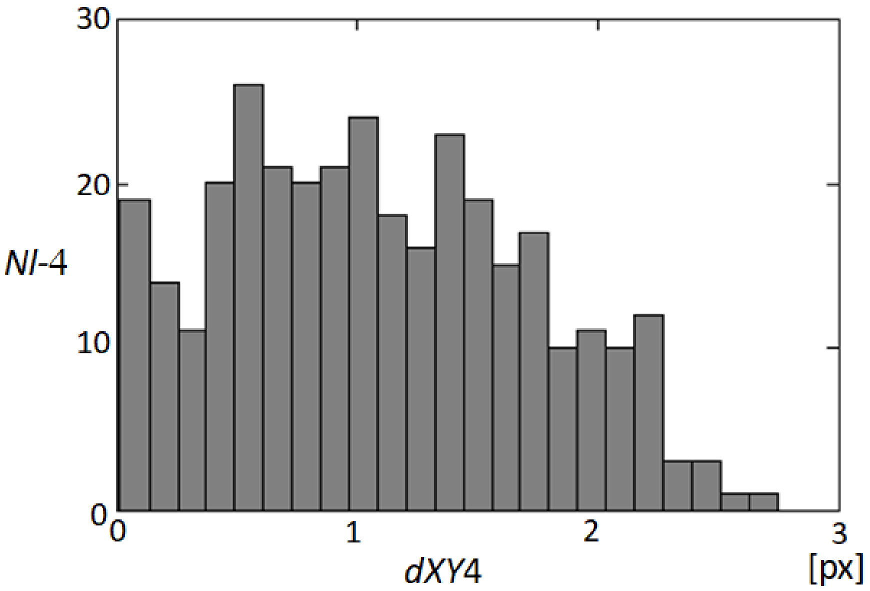

Figure 21. Histogram of lengths dXY4 (speeds) of fixational movements of point 4, recorded in the video sequence. Consistent with the observations depicted in Figure 10.

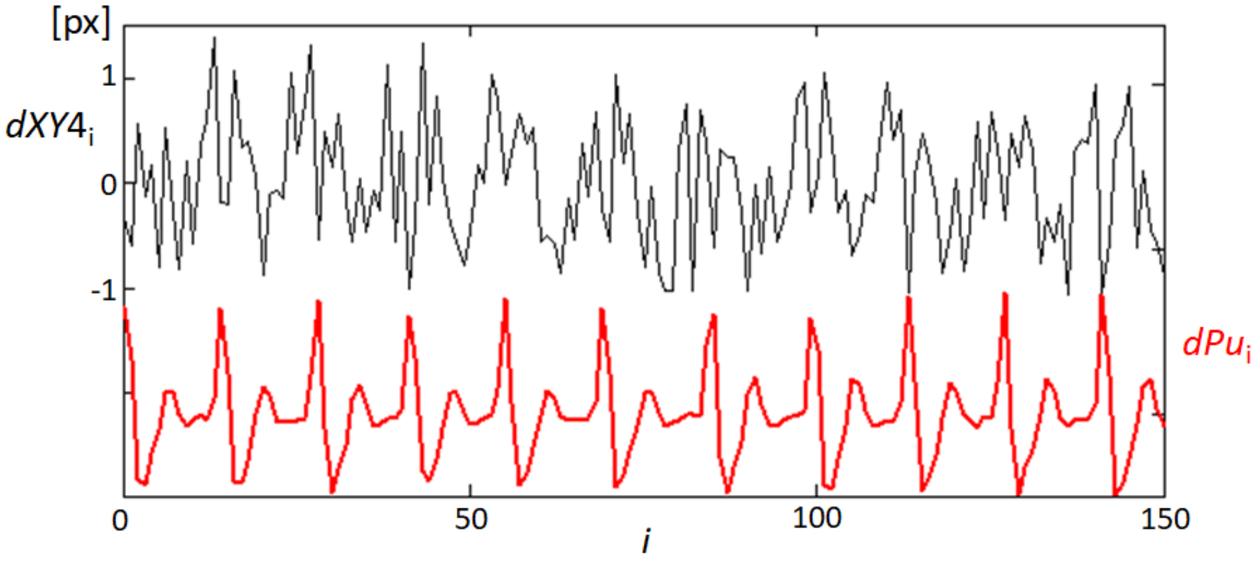

Figure 22. Time variability of lengths dXY4 of fixational eye movements of point 4 and the blood pulsation signal Pu for about half of the sequence. Similar to the patterns highlighted in Figure 11.

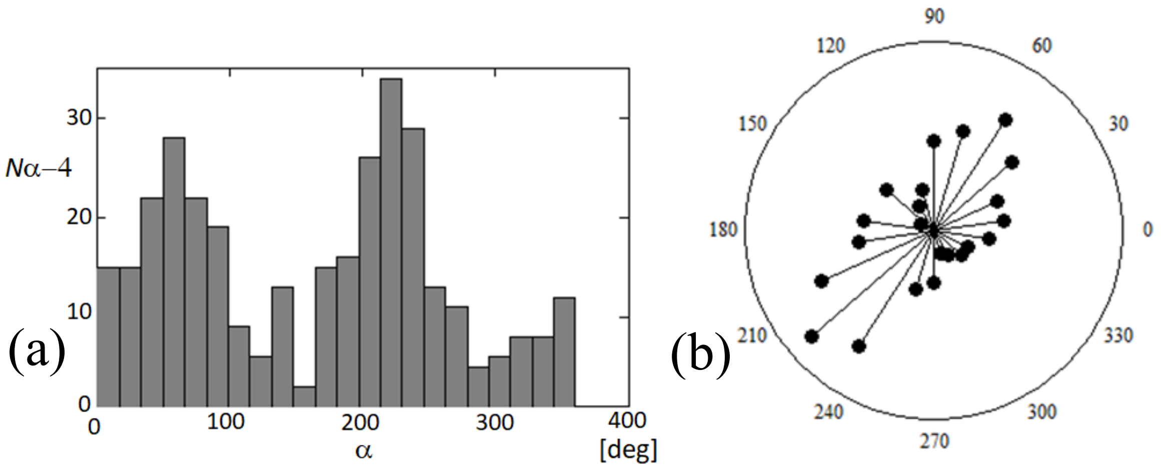

Figure 23. Histogram of orientation angles and eye movements in relation to the horizon line, a) on a rectangular plot, b) on a polar plot. In conformity with the data presented in Figure 12.

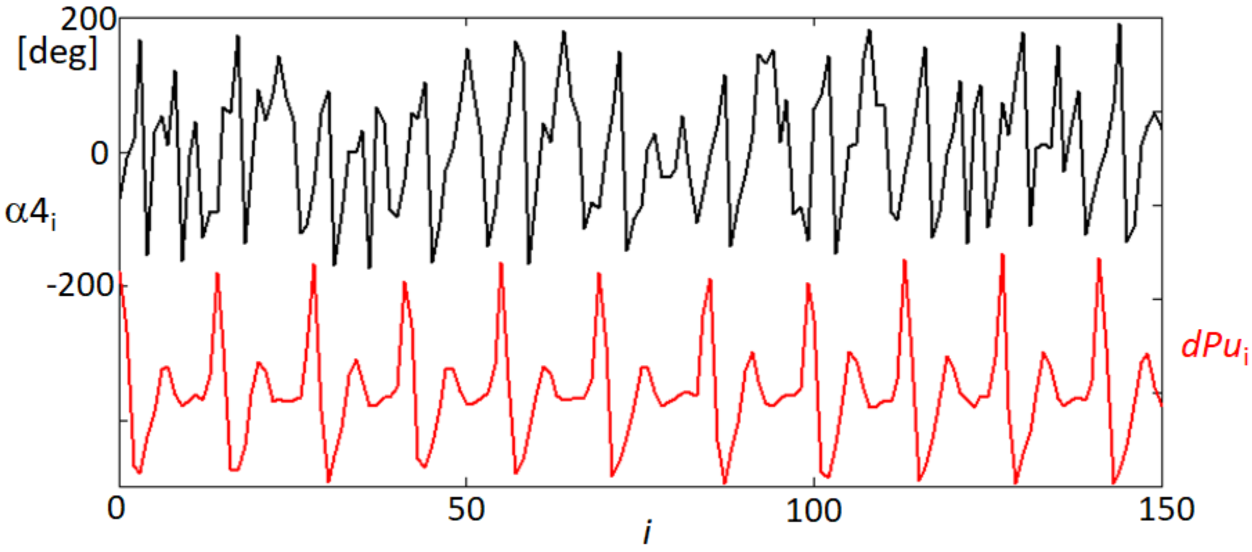

Figure 24. Time variability of the angle α of point 4 after linear detrend and blood pulsation signal Pu for about half of the recorded sequence. In line with the data presented in Figure 13.

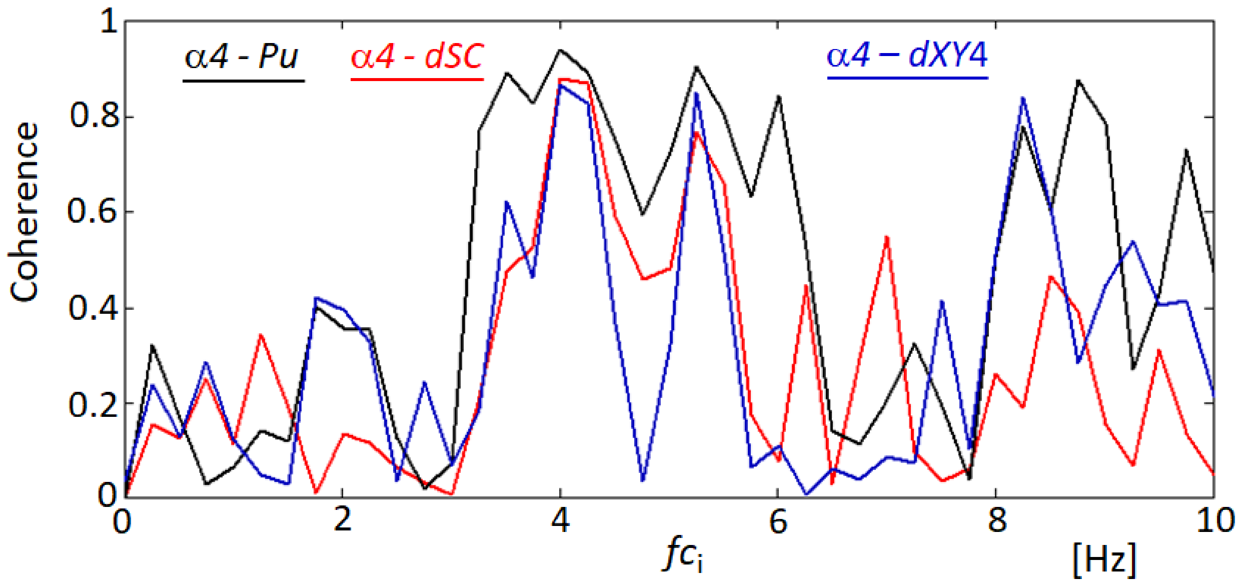

Figure 25. Coherence functions between orientation angle α4 and other examined parameters. Corresponding to the evidence shown in Figure 14.

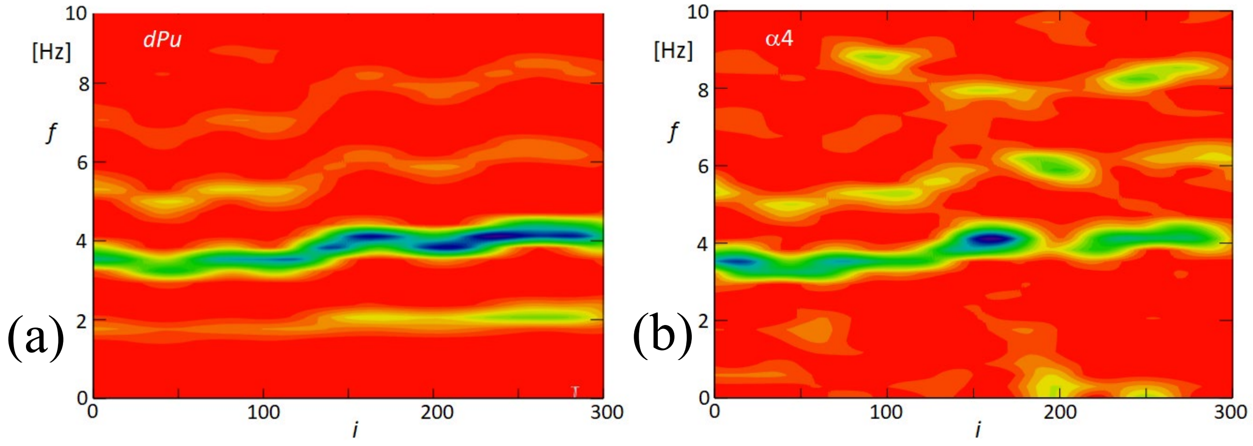

Figure 26. Time-frequency analysis of; a) blood pulsation derivative dPu, and b) variability of the angle α4.
